# Transition-Metal-Free One-Pot Synthesis of Fused Benzofuranamines and Benzo[*b*]thiophenamines

**DOI:** 10.3390/molecules28237738

**Published:** 2023-11-23

**Authors:** Ran Liu, Lili Lv, Bingchuan Yang, Ziyi Gu, Chenglong Li, Xueyan Lv, Chengcheng Ding, Xianqiang Huang, Dong Yuan

**Affiliations:** 1 School of Chemistry and Chemical Engineering, Liaocheng University, Liaocheng 252000, China; liuranbjt@163.com (R.L.); 19816255672@163.com (Z.G.); hxq@lcu.edu.cn (X.H.); 2 China Petroleum Planning and Engineering Institute, Dongying 257237, China; lvlili@petrochina.com.cn; 3 College of Chemistry and Chemical Engineering, Qilu Normal University, Jinan 250013, China; 4 School of Chemistry and Chemical Engineering, Shandong University, Jinan 250100, China; lxy17852267584@163.com (X.L.); 202220349@mail.sdu.edu.cn (C.D.); 5 The Department of Chemistry, University of South Florida, 4202 East Fowler Avenue, Tampa, FL 33620, USA; 6 Shandong Weijiao Holding Group Co., Ltd., Weifang 262404, China; lichenglong@sdcoke.com

**Keywords:** one-pot synthesis, transition metal-free, benzofuranamines, benzo[*b*]thiophenamines, C-C coupling

## Abstract

A series of benzofuran and benzo[*b*]thiophen derivatives was synthesized via a transition-metal-free one-pot process at room temperature. This one-pot protocol enables the synthesis of compounds with high reaction efficiency, mild conditions, simple methods, and a wide-ranging substrate scope. Regioselective five-membered heterocycles were constructed in good-to-excellent yields.

## 1. Introduction

**Scheme 1:**
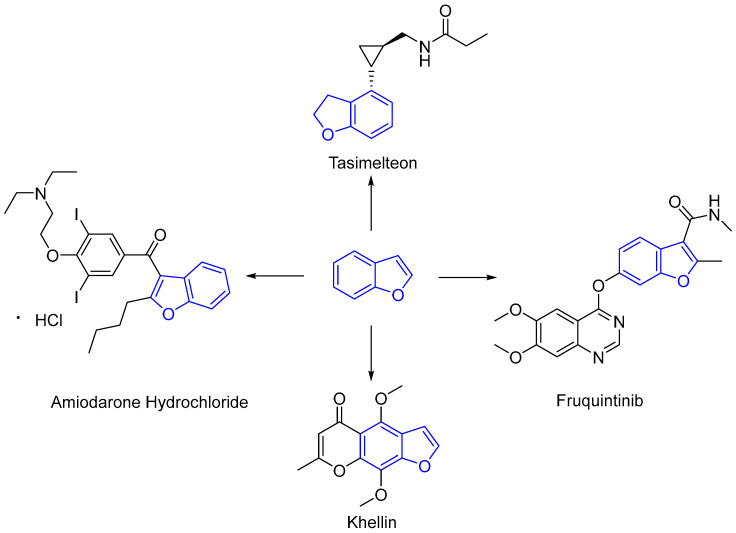
Structures of some biologically important benzofurans.

Benzofuran and benzo[*b*]thiophen derivatives have attracted considerable interest given their outstanding medicinal and biological properties [1,2]. Compounds with benzofuran functionalities have been widely employed to cure different kinds of diseases [3,4,5,6]. For example, Tasimelteon [7] is a small-molecule melatonin receptor agonist used to treat non-24-hour sleep disorders in patients with total blindness. Amiodarone hydrochloride [8,9] is a third class of antiarrhythmic drugs widely used for the treatment and prevention of arrhythmia and has a direct dilation effect on coronary arteries and peripheral vessels. Fruquintinib [10,11] can inhibit the formation of tumor neovascularization and eventually exert a tumor growth inhibition effect. It is a highly selective inhibitor of tumor angiogenesis. Khellin [12] is a micranochromone that has antiproliferative activity in vitro; meanwhile, it also has antispasmodic and coronary diastolic effects (Scheme 1). Fused benzofuran and benzothiophen also have antimicrobial, anti-inflammatory, antihypertensive, and analgesic activities [13].

**Scheme 2:**
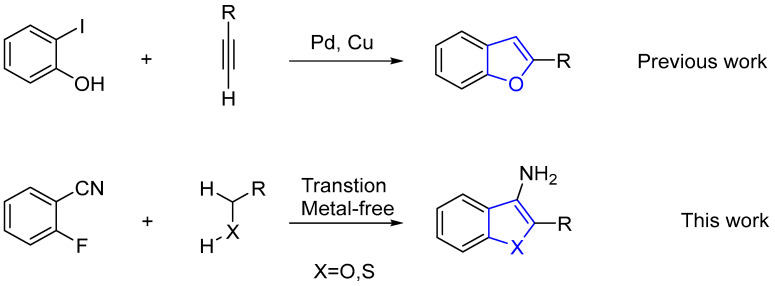
Attempts to synthesize benzofuran and benzothiophen derivatives in previous work and this work.

As a result, all kinds of methods have been developed for the construction of benzofuran and benzothiophen derivatives [14]. Classical methods for the synthesis of these derivatives are described in the literature. Zhang’s group [15] developed a palladium-catalyzed aryldifluoroalkylation method that involves the reaction of 1,6-enynes with ethyl difluoroiodoacetate and arylboronic acids, thereby achieving the desired derivatives. Jiangs’ group [16] developed a palladium-catalyzed fluoroalkylative cyclization of olefins with the formation of C_sp3_–CF_2_ and C–O/N bonds in one step to obtain difluoroalkylated 2,3-dihydrobenzofuran and indolin derivatives. In the synthesis of 2,3-disubstituted benzofuran, the Sonogashira coupling reaction [17] can be completed with a one-step reaction. It is prepared by coupling–cyclizing o-iodophenol with a terminal alkyne in the presence of powder potassium-fluoride-doped alumina and a mixture of powder palladium, cuprous iodide, and tri-phenylphosphine. However, these methods still suffer from some drawbacks, such as vigorous reaction conditions and multiple steps (Scheme 2). In this context, the development of a novel synthetic method to fulfill the atom economy and achieve great efficiency is highly desired [18,19,20].

**Scheme 3:**
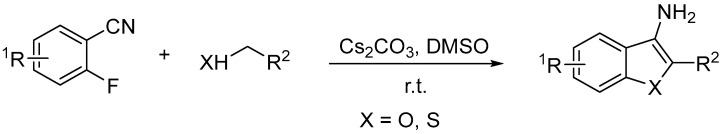
Synthesis of benzofuran-3-amines and benzo[*b*]thiophen-3-amines.

A one-step synthetic route would be a very useful improvement [21,22]. We focused on the development of the direct synthesis of heterocyclic systems using tandem reactions. Herein, we report an efficient and convergent one-pot synthetic strategy approach to benzofuran and benzothiophen derivatives under mild conditions. Benzofuran and benzothiophen scaffolds were obtained through the reaction of 2-fluorobenzonitriles and substituted alcohol at room temperature (Scheme 3).

## 2. Results

To obtain the optimized conditions, 2-fluorobenzonitrile, **1a**, and 1-hydroxypropan-2-one, **2a**, were chosen as models. As shown in Table 1, the reaction base, solvent, and time were investigated. The reaction proceeded with different bases in DMSO at room temperature, and Cs_2_CO_3_ provided the highest yields (Table 1, entry 5). In weak basic systems such as K_2_CO_3_ or K_3_PO_4_ at room temperature, no desired product, **3a**, was obtained (Table 1, entries 1,3). When we used the organic base Et_3_N in the reaction, no desired compound, **3a**, was detected either. In constructing **3a**, Cs_2_CO_3_ performed much better than KOH and *t*-BuOK, with a yield of 76% (Table 1, entries 5–7). The investigation of the solvent proved that the yields of the product in DMSO were higher than in CH_3_CN, THF, and DMF with the same base system (Table 1, entries 5, 9, 10, 11). Finally, we chose Cs_2_CO_3_ in DMSO as the most efficient system to accomplish the synthesis of the benzofuran derivatives, **3** (Table 1, entry 5).

**Figure 1:**
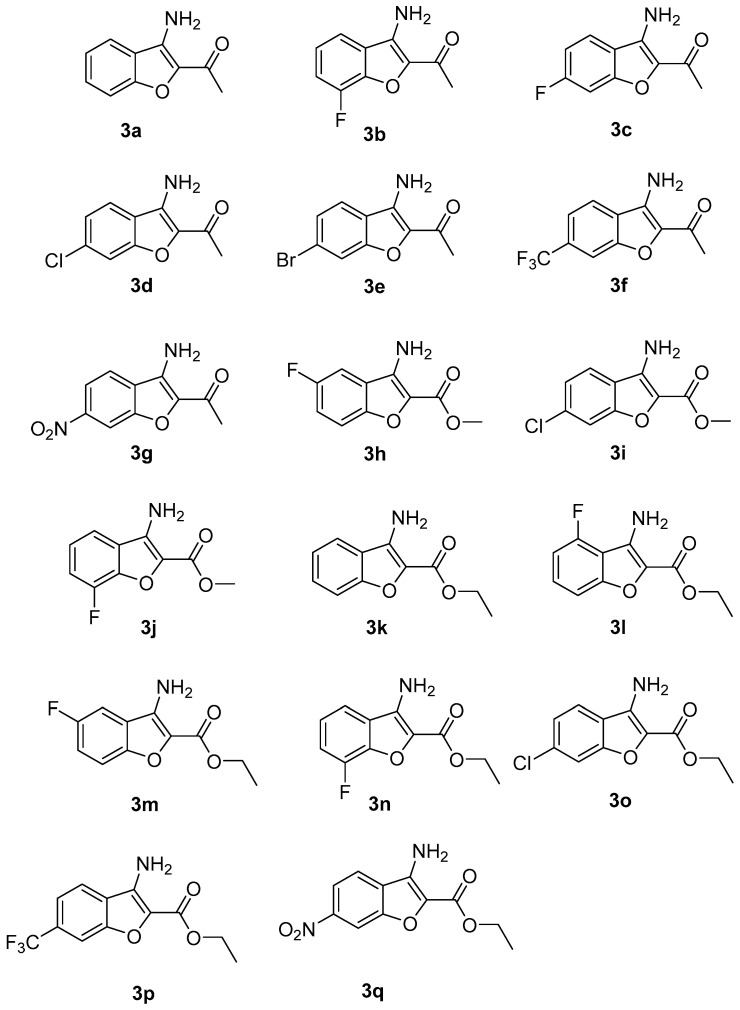
Structures of the desired compounds, **3a**–**3q**.

To explore the range of this methodology, various primary alcohols were studied (Table 2) under the selected reaction condition (Scheme 2). The structures of products **3a**–**3q** are shown in Figure 1. As shown in Table 2, both ketone and ester led to the formation of bicyclic products with high yields. However, 2-fluorobenzonitrile with strong electron-withdrawing groups (Table 2, entry 6, 7, 16, 17) obtained better yields than the non-substituted derivatives, and 2,4-difluorobenzonitrile obtained the best reaction yield of all the halogen-substituted scaffolds (Table 2, entry 3–5).

Additionally, a steric hindrance affected the reaction very slightly. Ethyl 2-hydroxyacetate (Table 2, entry 11–17) also obtained a high yield of **3**.

As shown in Table 3, a variety of substituted methanethiols, **4**, were used to expand the applicability of this methodology. Substituted 2-fluorobenzonitrile bearing methoxyl obtained a low yield of 31% in the reaction (Table 3, entry 9), whereas 2-fluorobenzonitrile with electron-withdrawing groups obtained higher yields (Table 3, entries 6–8). The steric hindrance affected the reaction very slightly, and butyl 2-hydroxyacetate also provided a high yield of **5** (Table 3, entry 4).

**Figure 2:**
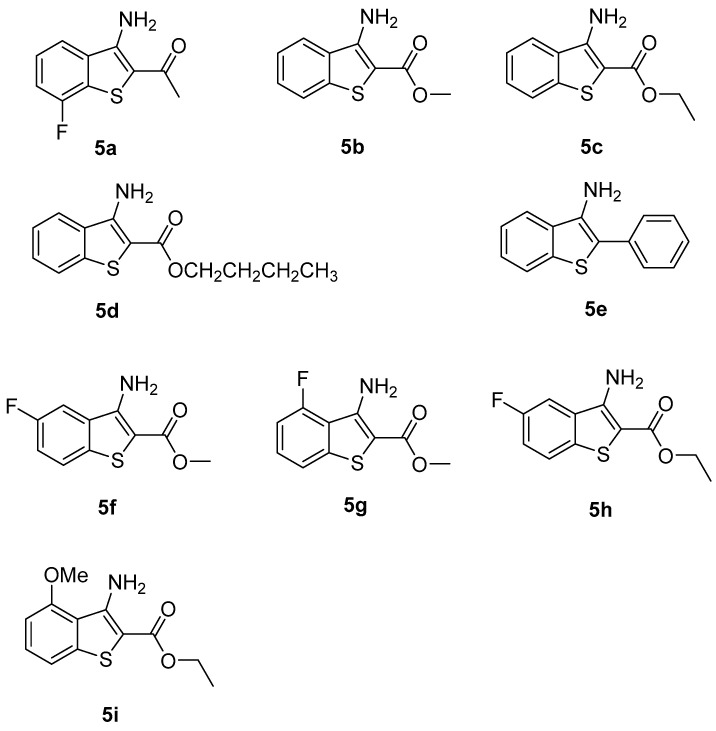
Structures of the desired compounds, **5a**–**5i**.

Aromatic substituted methanethiol also obtained a high yield of **5** (Table 3, entry 5). The structures of products **5a**–**5i** are shown in Figure 2.

**Scheme 4:**
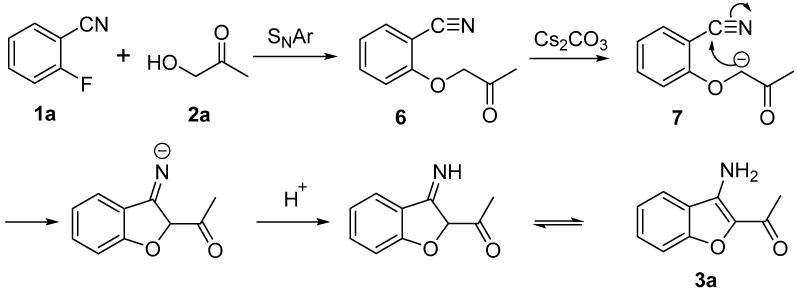
Plausible mechanism of the formation of **3a**.

Based on our previous work [23], a plausible reaction mechanism is presented in Scheme 4. Compounds **1a** and **2a** undergo nucleophilic aromatic substitution, providing compound **6**. In the presence of Cs_2_CO_3_, the carbanion, **7**, that forms attacks the nitrile group, leading to cyclization and imine anion formation. Then, proton addition and tautomerism lead to the corresponding product, **3a**.

**Figure 3:**
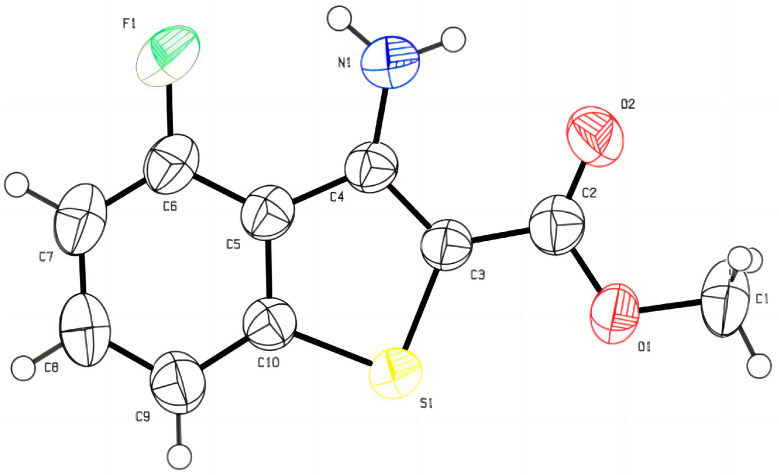
X-ray structure of compound **5g**.

To demonstrate the structure of benzothiophene, the molecular configuration of product **5g** was determined through X-ray crystallographic analysis (Figure 3).

## 3. Experimental Section 

### 3.1. General

^1^H and ^13^C NMR spectra were recorded with a 300 spectrometer or a 400 spectrometer in CDCl_3_. HRMS spectra were determined with a Q-TOF spectrograph. Compounds **3a**–**3q** and **5a**–**5i** were prepared according to the literature. Other reagents (Adamas) were commercially available and were used without further purification. All reactions were monitored via thin-layer chromatography (TLC). For the NMR spectrum of compounds see the Supplementary Materials.

### 3.2. General Experimental Procedure for 1-(3-Aminobenzofuran-2-yl)ethan-1-one (***3a***)

To a solution of DMSO (15 mL), 2-fluorobenzonitrile, **1a** (0.12 g, 1.0 mmol); propionic acid, **2a** (0.037 g, 0.5 mmol); and Cs_2_CO_3_ (0.98 g, 3.0 mmol) were added and stirred for 1 h at room temperature; then, more propionic acid, **2a** (0.037 g, 0.5 mmol), was added and stirred at r.t. over 4 h. Brine (40 mL) was poured into the solution, and the mixture was extracted with CH_2_Cl_2_ (3 × 40 mL). The organic layers were combined and dried by over-anhydrous Na_2_SO_4_. The product was purified via flash chromatography on silica gel (Hexane/EtOAc = 5:1). Compound **3a** was obtained as a white solid (mp: 175–176 °C, 0.13 g, 76% yield). ^1^H NMR (CDCl_3_, 300 MHz): δ 7.59–7.56 (m, 1H), 7.52–7.46 (m, 1H), 7.42–7.40 (d, *J* = 8.1 Hz, 1H), 7.26–7.21 (m, 1H), 5.59 (s, 2H), 2.50 (s, 3H); ^13^C NMR (CDCl_3_, 75 MHz): δ 189.8, 154.0, 138.5, 135.4, 129.4, 122.2, 121.3, 120.3, 112.6, 25.9; FT-HRMS (ESI) calcd for C_10_H_9_NO_2_ [(M + H)^+^]: 176.0667; found, 176.0691.

### 3.3. General Experimental Procedure for 1-(3-Amino-7-fluorobenzo[b]thiophen-2-yl)ethan-1-one (***5a***)

This compound was prepared in the same way as described for **3a** by using 2,3-difluorobenzonitrile (0.14 g, 1.0 mmol), 1,2-difluoro-4-nitrobenzene **4a** (0.09 g, 1.0 mmol), and Cs_2_CO_3_ (0.98 g, 3.0 mmol) in DMSO (15 mL) at room temperature. The product was purified via flash chromatography on silica gel (hexane/EtOAc = 5:1) to obtain **5a** (mp: 245–246 °C, 0.16 g, 78% yield) as a pale yellow solid. ^1^H NMR (CDCl_3_, 400 MHz): δ 7.88 (s, 1H), 7.48 (t, *J* = 8.8 Hz, 2H), 5.88 (s, 2H), 3.89 (s, 3H); ^13^C NMR (CDCl_3_, 75 MHz): δ 165.6, 147.9, 141.3, 128.8 (*J*_C,F_ = 267 Hz), 125.9, 122.4 (*J*_C,F_ = 22 Hz), 51.4; FT-HRMS (ESI) calcd for C_10_H_8_FNOS [(M + H)^+^]: 210.0344; found, 210.0567.

### 3.4. X-ray Crystal Structure Analysis of Compound ***5g*** [24,25]

Single crystals of **5g** suitable for X-ray crystal analysis were obtained via recrystallization from a hexane/CH_2_Cl_2_ mixed solvent. Intensity data were collected at 293 K with an X-ray diffractometer with Mo Kα radiation (*λ* = 0.71073 Å) and a graphite monochrometer. A total of 1137 reflections were measured at a maximum 2*θ* angle of 50.0°, of which 1781 were independent reflections (*R*_int_ = 0.0406). The structure was determined with direct methods (SHELXS-97)^22^ and refined by using full-matrix least-squares on *F*^2^ (SHELEXL-97)^22^. The crystal data are as follows: C_10_H_8_FNO_2_S; F_W_ = 225.03; crystal size, 0.12 × 0.10 × 0.10 mm^3^; monoclinic, P21/c, *a* = 13.7929(8) Å, *b* = 3.9422(3) Å; *c* = 22.5228(15) Å; *V* = 991.90(12) Å^3^; Z = 4. The refinement converged to *R*_1_ = 0.0637, *wR*_2_ = 0.1604 (*I* > 2σ(*I*)).

### 3.5. Characterization Data

1-(3-Amino-7-fluorobenzofuran-2-yl)ethan-1-one (**3b**): 155 mg (80% yield), white solid, mp: 188–189 °C; ^1^H NMR (CDCl_3_, 300 MHz): δ 7.35 (dd, *J* = 1.5, 7.5 Hz, 1H), 7.25–7.14 (m, 2H), 5.31 (s, 1H), 2.53 (s, 3H); ^13^C NMR (CDCl_3_, 75 MHz): δ 190.0, 148.6 (*J*_C,F_ = 249 Hz), 141.4 (*J*_C,F_ = 13 Hz), 138.1, 136.1, 124.8, 122.8 (*J*_C,F_ = 6 Hz), 115.7, 115.1 (*J*_C,F_ = 16 Hz), 25.97; FT-HRMS (ESI) calcd for C_10_H_8_FNO_2_ [(M + H)^+^]: 194.0573; found, 194.0625.

1-(3-Amino-6-fluorobenzofuran-2-yl)ethan-1-one (**3c**): 158 mg (82% yield), white solid, mp: 188–189 °C; ^1^H NMR (CDCl_3_, 400 MHz): δ 7.56 (t, *J* = 8.0 Hz, 1H), 6.79–6.71 (m, 2H), 4.70 (s, 2H), 3.83 (s, 3H); ^13^C NMR (CDCl_3_, 100 MHz): δ 163.1, 159.7 (*J*_C,F_ = 257 Hz), 157.9 (*J*_C,F_ = 11 Hz), 129.70, 109.3, 106.7, 98.5 (*J*_C,F_ = 23 Hz), 89.6 (*J*_C,F_ = 16 Hz), 26.0; FT-HRMS (ESI) calcd for C_10_H_8_FNO_2_ [(M + H)^+^]: 194.0573; found, 194.0653

1-(3-Amino-6-chlorobenzofuran-2-yl)ethan-1-one (**3d**): 163 mg (78% yield), white solid, mp: 217–219 °C; ^1^H NMR (CDCl_3_, 400 MHz): δ 7.50 (d, *J* = 8.4 Hz, 1H), 7.44 (d, *J* = 1.6 Hz, 1H), 7.24 (dd, *J* = 1.6, 8.4 Hz, 1H), 5.58 (s, 2H), 2.50 (s, 3H); ^13^C NMR (CDCl_3_, 100 MHz): δ 189.7, 153.9, 137.8, 135.4, 123.3, 120.9, 120.0, 113.1, 110.0, 25.9; FT-HRMS (ESI) calcd for C_10_H_8_ClNO_2_ [(M + H)^+^]: 211.0214; found, 211.0265.

1-(3-Amino-6-bromobenzofuran-2-yl)ethan-1-one (**3e**): 142 mg (56% yield), white solid, mp: 247–249 °C; ^1^H NMR (CDCl_3_, 400 MHz): δ 7.61 (d, *J* = 1.2 Hz, 1H), 7.45 (d, *J* = 8.4 Hz, 1H), 7.37 (dd, *J* = 1.6, 8.4 Hz, 1H), 5.56 (s, 2H), 2.49 (s, 3H); ^13^C NMR (CDCl_3_, 100 MHz): δ 189.8, 154.0, 137.8, 135.6, 125.9, 123.1, 121.1, 120.3, 116.1, 26.0; FT-HRMS (ESI) calcd for C_10_H_8_BrNO_2_ [(M + H)^+^]: 254.9718; found, 254.9816.

1-(3-Amino-6-(trifluoromethyl)benzofuran-2-yl)ethan-1-one (**3f**): 219 mg (90% yield), white solid, mp: 203–204 °C; ^1^H NMR (CDCl_3_, 400 MHz): δ 7.70 (d, *J* = 8.8 Hz, 2H), 7.50 (d, *J* = 8.4 Hz, 1H), 5.60 (s, 2H), 2.53 (s, 3H); ^13^C NMR (CDCl_3_, 100 MHz): δ 190.3, 152.8, 137.1, 136.6, 131.5 (*J*_C,F_ = 32 Hz), 130.8 (*J*_C,F_ = 33 Hz) 125.3, 124.1, 122.6, 119.0 (*J*_C,F_ = 3 Hz), 110.3 (*J*_C,F_ = 4 Hz), 26.1; FT-HRMS (ESI) calcd for C_11_H_8_F_3_NO_2_ [(M + H)^+^]: 244.0541; found, 244.0645.

1-(3-Amino-6-nitrobenzofuran-2-yl)ethan-1-one (**3g**): 178 mg (81% yield), white solid, mp none; ^1^H NMR (CDCl_3_, 400 MHz): δ 8.35 (d, *J* = 1.6 Hz, 1H), 8.16 (dd, *J* = 2.0, 8.4 Hz, 1H), 7.72 (d, *J* = 8.8 Hz, 1H), 5.59 (s, 2H), 2.56 (s, 3H); ^13^C NMR (CDCl_3_, 100 MHz): δ 190.5, 152.3, 148.3, 138.2, 136.5, 126.4, 120.6, 117.5, 109.1, 26.2; FT-HRMS (ESI) calcd for C_10_H_8_N_2_O_4_ [(M + H)^+^]: 221.0518; found, 221.0589.

Methyl 3-amino-5-fluorobenzofuran-2-carboxylate (**3h**): 161 mg (77% yield), white solid, mp: 180–182 °C; ^1^H NMR (CDCl_3_, 400 MHz): δ 7.40–7.34 (m, 1H), 7.23 (d, *J* = 8.0 Hz, 1H), 6.90–6.86 (m, 1H), 5.21 (s, 2H), 3.96 (s, 3H); ^13^C NMR (CDCl_3_, 100 MHz): δ 163.4, 161.5, 157.3 (*J*_C,F_ = 250 Hz), 129.4 (*J*_C,F_ = 9 Hz), 111.1 (*J*_C,F_ = 18 Hz), 108.7 (*J*_C,F_ = 5 Hz), 107.8 (*J*_C,F_ = 18 Hz), 51.5; FT-HRMS (ESI) calcd for C_10_H_8_FNO_3_ [(M + H)^+^]: 210.0522; found, 210.0539.

Methyl 3-amino-6-chlorobenzofuran-2-carboxylate (**3i**): 173 mg (72% yield), white solid, mp: 210–211 °C; ^1^H NMR (CDCl_3_, 400 MHz): δ 7.49–7.45 (m, 2H), 7.24 (dd, *J* = 1.6, 8.4 Hz, 1H), 4.99 (s, 2H), 3.97 (s, 3H); ^13^C NMR (CDCl_3_, 100 MHz): δ 161.6, 153.9, 138.2, 134.9, 125.9, 123.3, 120.4, 120.2, 113.0, 110.0, 103.1, 51.6; FT-HRMS (ESI) calcd for C_10_H_8_ClNO_3_ [(M + H)^+^]: 227.0163; found, 227.0195.

Methyl 3-amino-7-fluorobenzofuran-2-carboxylate (**3j**): 164 mg (78% yield), white solid, mp: 180–182 °C; ^1^H NMR (CDCl_3_, 400 MHz): δ 7.35–7.32 (m, 1H), 7.22–7.17 (m, 2H), 5.02 (s, 2H), 3.97 (s, 3H); ^13^C NMR (CDCl_3_, 100 MHz): δ 161.7, 148.4 (*J*_C,F_ = 250 Hz), 141.4 (*J*_C,F_ = 13 Hz), 138.6, 126.2, 125.0 (*J*_C,F_ = 3 Hz), 123.0 (*J*_C,F_ = 6 Hz), 115.2 (*J*_C,F_ = 4 Hz), 114.7 (*J*_C,F_ = 16 Hz), 51.6; FT-HRMS (ESI) calcd for C_10_H_8_FNO_3_ [(M + H)^+^]: 210.0522; found, 210.0598.

Ethyl 3-aminobenzofuran-2-carboxylate (**3k**): 146 mg (71% yield), white solid, mp: 179–180 °C; ^1^H NMR (CDCl_3_, 300 MHz): δ 7.57–7.54 (m, 1H), 7.46–7.44 (m, 2H), 7.27–7.22 (m, 2H), 4.96 (s, 1H), 4.45 (q, *J* = 6.9 Hz, 2H), 1.44 (t, *J* = 6.9 Hz, 3H); ^13^C NMR (CDCl_3_, 75 MHz): δ 161.67, 154.02, 130.91, 128.85, 128.77, 125.56, 122.30, 121.67, 119.59, 112.66, 65.58, 60.46, 30.59, 29.71, 19.19, 14.66, 13.72; FT-HRMS (ESI) calcd for C_11_H_11_NO_3_ [(M + H)^+^]: 206.0772; found, 206.0785.

Ethyl 3-amino-4-fluorobenzofuran-2-carboxylate (**3l**): 162 mg (73% yield), white solid, mp: 192–193 °C; ^1^H NMR (CDCl_3_, 400 MHz): δ 7.39–7.34 (m, 1H), 7.24 (d, *J* = 8.4 Hz, 1H), 6.88 (dd, *J* = 8.0, 9.6 Hz, 1H), 5.19 (s, 2H), 4.44 (q, *J* = 7.2 Hz, 2H), 1.44 (t, *J* = 7.2 Hz, 3H); ^13^C NMR (CDCl_3_, 100 MHz): δ 161.4, 157.3 (*J*_C,F_ = 250 Hz), 155.2, 129.3 (*J*_C,F_ = 7 Hz), 111.1 (*J*_C,F_ = 20 Hz), 108.8 (*J*_C,F_ = 5 Hz), 107.7, (*J*_C,F_ = 18 Hz), 60.5, 14.6; FT-HRMS (ESI) calcd for C_11_H_10_FNO_3_ [(M + H)^+^]: 224.0678; found, 224.0693.

Ethyl 3-amino-5-fluorobenzofuran-2-carboxylate (**3m**): 179 mg (80% yield), white solid, mp: 192–193 °C; ^1^H NMR (CDCl_3_, 400 MHz): δ 7.44 (d, *J* = 8.0 Hz, 1H), 7.37–7.32 (m, 1H), 6.94 (dd, *J* = 8.0, 9.6 Hz, 1H), 6.31 (s, 2H), 4.34 (q, *J* = 7.2 Hz, 2H), 1.38 (t, *J* = 7.2 Hz, 3H); ^13^C NMR (CDCl_3_, 100 MHz): δ 165.2, 159.6 (*J*_C,F_ = 250 Hz), 147.6, 142.1, 128.8 (*J*_C,F_ = 9 Hz), 120.3 (*J*_C,F_ = 14 Hz), 119.2 (*J*_C,F_ = 4 Hz), 109.3 (*J*_C,F_ = 20 Hz), 97.6, 60.4, 14.49; FT-HRMS (ESI) calcd for C_11_H_10_FNO_3_ [(M + H)^+^]: 224.0678; found, 224.0695.

Ethyl 3-amino-7-fluorobenzofuran-2-carboxylate (**3n**): 181 mg (81% yield), white solid, mp: 192–193 °C; ^1^H NMR (CDCl_3_, 400 MHz): δ 7.34–7.32 (m, 1H), 7.22–7.17 (m, 2H), 5.00 (s, 2H), 4.45 (q, *J* = 7.2 Hz, 2H), 1.44 (t, *J* = 7.2 Hz, 3H); ^13^C NMR (CDCl_3_, 100 MHz): δ 161.4, 148.4 (*J*_C,F_ = 250 Hz), 141.4 (*J*_C,F_ = 13 Hz), 138.4, 126.5, 125.1 (*J*_C,F_ = 3 Hz), 122.8 (*J*_C,F_ = 6 Hz), 115.1 (*J*_C,F_ = 4 Hz), 114.5 (*J*_C,F_ = 15 Hz), 60.6, 14.5; FT-HRMS (ESI) calcd for C_11_H_10_FNO_3_ [(M + H)^+^]: 224.0678; found, 224.0689.

Ethyl 3-amino-6-chlorobenzofuran-2-carboxylate (**3o**): 177 mg (74% yield), white solid, mp: 221–222 °C; ^1^H NMR (CDCl_3_, 400 MHz): δ 7.47 (d, *J* = 8.8 Hz, 2H), 7.24 (dd, *J* = 2.0, 8.4 Hz, 1H), 4.97 (s, 2H), 4.44 (q, *J* = 7.2 Hz, 2H), 1.44 (t, *J* = 7.2 Hz, 3H); ^13^C NMR (CDCl_3_, 100 MHz): δ 161.4, 153.9, 134.7, 123.3, 120.3, 113.0, 60.6, 14.6; FT-HRMS (ESI) calcd for C_11_H_10_ClNO_3_ [(M + H)^+^]: 241.0320; found.

Ethyl 3-amino-6-(trifluoromethyl)benzofuran-2-carboxylate (**3p**): 246 mg (90% yield), white solid, mp: 207–208 °C; ^1^H NMR (CDCl_3_, 400 MHz): δ 7.73 (s, 1H), 7.68 (d, *J* = 8.4 Hz, 1H), 7.50 (d, *J* = 8.4 Hz, 1H), 5.02 (s, 2H), 4.46 (q, *J* = 7.2 Hz, 2H), 1.45 (t, *J* = 7.2 Hz, 3H); ^13^C NMR (CDCl_3_, 100 MHz): δ 161.3, 152.8, 137.5, 131.1, 130.8, 130.3 (*J*_C,F_ = 30 Hz), 128.0, 127.3, 125.3, 124.4, 122.6, 120.4, 119.1 (*J*_C,F_ = 3 Hz), 110.2 (*J*_C,F_ = 4 Hz), 60.8, 14.6; FT-HRMS (ESI) calcd for C_12_H_10_F_3_NO_3_ [(M + H)^+^]: 274.0646; found, 274.0686.

Ethyl 3-amino-6-nitrobenzofuran-2-carboxylate (**3q**): 218 mg (87% yield), white solid, mp none; ^1^H NMR (CDCl_3_, 400 MHz): δ 7.68 (d, J = 1.2 Hz, 1H), 7.52 (d, *J* = 8.4 Hz, 1H), 7.20 (dd, *J* = 1.6, 8.4 Hz, 1H), 5.88 (s, 2H), 4.35 (q, *J* = 7.2 Hz, 2H), 1.38 (t, *J* = 7.2 Hz, 3H); ^13^C NMR (CDCl_3_, 100 MHz): δ 165.2, 162.6, 147.7, 140.9, 134.4, 129.8, 124.7, 122.9, 122.0, 99.8, 60.6, 14.5; FT-HRMS (ESI) calcd for C_11_H_10_N_2_O_5_ [(M + H)^+^]: 251.0623; found, 251.0635.

Methyl 3-aminobenzo[*b*]thiophene-2-carboxylate (**5b**): 157 mg (76% yield), pale yellow solid, mp: 224–225 °C; ^1^H NMR (CDCl_3_, 300 MHz): δ 7.73 (d, *J* = 8.1 Hz, 1H), 7.63 (d, *J* = 7.8 Hz, 1H), 7.49–7.44 (m, 1H), 7.39–7.34 (m, 1H), 5.80 (s, 2H), 3.89 (s, 3H); ^13^C NMR (CDCl_3_, 75 MHz): δ 165.9, 148.5, 140.0, 131.3, 128.2, 123.9, 123.4, 121.2, 99.0, 51.5; FT-HRMS (ESI) calcd for C_10_H_9_NO_2_S [(M + H)^+^]: 208.0388; found, 208.0398.

Ethyl 3-aminobenzo[*b*]thiophene-2-carboxylate (**5c**): 164 mg (74% yield), pale yellow solid, mp: 235–237 °C; ^1^H NMR (CDCl_3_, 300 MHz): δ 7.72 (d, *J* = 8.1 Hz, 1H), 7.63 (d, *J* = 8.1 Hz, 1H), 7.49–7.43 (m, 1H), 7.39–7.34 (m, 1H), 5.61 (s, 1H), 4.36 (q, *J* = 7.2 Hz, 2H), 1.39 (t, *J* = 7.2 Hz, 3H); ^13^C NMR (CDCl_3_, 75 MHz): δ 165.6, 148.3, 140.0, 131.5, 128.1, 123.8, 123.4, 121.2, 99.5, 60.4, 14.5; FT-HRMS (ESI) calcd for C_11_H_11_NO_2_S [(M + H)^+^]: 222.0544; found, 222.0609.

Butyl 3-aminobenzo[*b*]thiophene-2-carboxylate (**5d**): 192 mg (77% yield), pale yellow solid, mp: 258–259 °C; ^1^H NMR (CDCl_3_, 300 MHz): δ 7.73–7.66 (m, 2H), 7.48–7.42 (m, 1H), 7.38–7.33 (m, 1H), 5.26 (s, 2H), 4.30 (t, *J* = 6.6 Hz, 2H), 1.80–––1.69 (m, 2H), 1.54–1.41 (m, 2H), 0.98 (t, *J* = 7.5 Hz, 3H); ^13^C NMR (CDCl_3_, 75 MHz): δ 165.6, 147.9, 140.0, 131.5, 128.2, 123.9, 123.4, 121.2, 100.0, 64.3, 30.9, 19.3, 13.8; FT-HRMS (ESI) calcd for C_13_H_15_NO_2_S [(M + H)^+^]: 250.0857; found, 250.0903.

2-Phenylbenzo[*b*]thiophen-3-amine (**5e**): 191 mg (85% yield), pale yellow solid, mp: 253–255 °C; ^1^H NMR (CDCl_3_, 300 MHz): δ 7.78 (dd, *J* = 1.5, 6.9 Hz, 1H), 7.64–7.57 (m, 3H), 7.49–7.44 (m, 2H), 7.42–7.29 (m, 3H), 4.07 (s, 2H); ^13^C NMR (CDCl_3_, 75 MHz): δ 137.6, 134.5, 134.3, 133.6, 129.2, 128.4, 127.0, 124.8, 123.9, 122.7, 119.9, 115.6; FT-HRMS (ESI) calcd for C_14_H_11_NS [(M + H)^+^]: 226.0646; found, 226.0649.

Methyl 3-amino-5-fluorobenzo[*b*]thiophene-2-carboxylate (**5f**): 169 mg (75% yield), pale yellow solid, mp: 237–238 °C; ^1^H NMR (CDCl_3_, 400 MHz): δ 7.67 (dd, *J* = 4.8, 8.8 Hz, 1H), 7.30 (dd, *J* = 2.4, 8.8 Hz, 1H), 7.25–7.21 (m, 1H), 5.83 (s, 2H), 3.90 (s, 3H); ^13^C NMR (CDCl_3_, 100 MHz): δ 165.6, 160.4 (*J*_C,F_ = 242 Hz), 147.8, 136.4, 135.2, 132.2 (*J*_C,F_ = 8 Hz), 130.7, 124.8 (*J*_C,F_ = 8 Hz), 117.2 (*J*_C,F_ = 25 Hz), 109.9, 106.9 (*J*_C,F_ = 23 Hz), 101.2, 51.7; FT-HRMS (ESI) calcd for C_10_H_8_FNO_2_S [(M + H)^+^]: 226.0293; found, 226.0356.

Methyl 3-amino-4-fluorobenzo[*b*]thiophene-2-carboxylate (**5g**): 171 mg (76% yield), pale yellow solid, mp: 237–238 °C; ^1^H NMR (CDCl_3_, 400 MHz): δ 7.44 (d, *J* = 8.0 Hz, 1H), 7.37–7.32 (m, 1H), 7.17 (t, *J* = 8.8 Hz, 1H), 5.92 (s, 2H), 3.90 (s, 3H); ^13^C NMR (CDCl_3_, 100 MHz): δ 165.6, 157.9 (*J*_C,F_ = 246 Hz), 148.3, 134.5 (*J*_C,F_ = 10 Hz), 127.1 (*J*_C,F_ = 20 Hz), 125.4 (*J*_C,F_ = 7 Hz), 117.1, 117.0, 113.2 (*J*_C,F_ = 18 Hz), 99.9, 53.4, 51.7; FT-HRMS (ESI) calcd for C_10_H_8_FNO_2_S [(M + H)^+^]: 226.0293; found, 226.0348.

Ethyl 3-amino-5-fluorobenzo[*b*]thiophene-2-carboxylate (**5h**): 179 mg (75% yield), pale yellow solid, mp: 249–250 °C; ^1^H NMR (CDCl_3_, 400 MHz): δ 7.67 (dd, *J* = 4.8, 8.8 Hz, 1H), 7.30 (dd, *J* = 2.0, 8.8 Hz, 1H), 7.25–7.21 (m, 1H), 5.80 (s, 2H), 4.36 (q, *J* = 7.2 Hz, 2H), 1.40 (t, *J* = 7.2 Hz, 3H); ^13^C NMR (CDCl_3_, 100 MHz): δ 165.3, 160.4 (*J*_C,F_ = 242 Hz), 147.6, 135.2, 132.3 (*J*_C,F_ = 8 Hz), 124.8 (*J*_C,F_ = 9 Hz), 117.1 (*J*_C,F_ = 25 Hz), 106.9 (*J*_C,F_ = 23 Hz), 60.6, 14.5; FT-HRMS (ESI) calcd for C_11_H_10_FNO_2_S [(M + H)^+^]: 240.0450; found, 240.0468.

Ethyl 3-amino-4-methoxybenzo[*b*]thiophene-2-carboxylate (**5i**): 78 mg (31% yield), pale yellow solid, mp: 282–283 °C; ^1^H NMR (CDCl_3_, 400 MHz): δ 7.37–7.28 (m, 1H), 7.24 (d, *J* = 4.0 Hz, 1H), 6.75 (s, 2H), 6.71–6.64 (m, 1H), 4.32 (q, *J* = 8.0 Hz, 2H), 2.62 (s, 3H), 1.37 (t, *J* = 8.0 Hz, 3H); ^13^C NMR (CDCl_3_, 100 MHz): δ 165.5, 157.7, 148.8, 142.0, 129.0, 120.9, 115.8, 104.0, 60.0, 55.6, 14.5; FT-HRMS (ESI) calcd for C_12_H_13_NO_3_S [(M + H)^+^]: 252.0650; found, 252.0687.

## 4. Conclusions

In conclusion, a variety of benzofuran and benzo[*b*]thiophen derivatives was synthesized in good-to-excellent yields via Smiles rearrangement. At room temperature, an efficient and simple method was used to construct regioselective five-membered heterocycles. The outstanding features of this approach are mild conditions and transition-metal-free one-pot synthesis. This green and clean synthetic methodology has potential applications in the synthesis of biologically and medicinally relevant compounds. Our team is currently conducting more research to widen the applications of this technology.

## Figures and Tables

**Table 1:** Optimization of conditions *^a^*.

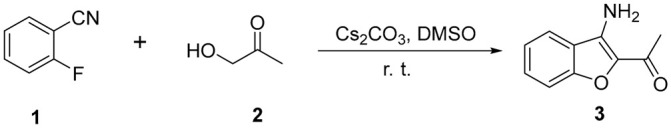					
**Entry**	**Base**	**Solvent**	**T (°C)**	**Time (h)**	**Yield (%) *^b^***
1	K_2_CO_3_	DMSO	r.t.	4	n.d.
2	K_2_CO_3_	DMSO	60	6	6
3	K_3_PO_4_	DMSO	r.t.	4	n.d.
4	K_3_PO_4_	DMSO	60	6	18
5	Cs_2_CO_3_	DMSO	r.t.	4	76
6	KOH	DMSO	r.t.	4	36
7	*t*-BuOK	DMSO	r.t.	1	56
8	Et_3_N	DMSO	r.t.	5	n.d.
9	Cs_2_CO_3_	THF	r.t.	4	n.d.
10	Cs_2_CO_3_	CH_3_CN	r.t.	4	n.d.
11	Cs_2_CO_3_	DMF	r.t.	4	n.d.

*^a^* Reaction conditions: 2-fluorobenzonitrile, **1a** (1.0 equiv.); 1-hydroxypropan-2-one, **2a** (1.0 equiv.); base (3.0 equiv.). *^b^* Isolated yields. n.d.: not detected.

**Table 2:** Synthesis of benzofuran-3-amines *^a^.*

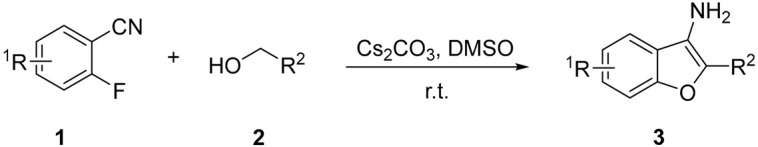					
**Entry**	**R^1^**	**R^2^**	**Time (h)**	**Product**	**Yield (%) *^b^***
1	H	-COCH_3_	6	**3a**	76
2	3-F	-COCH_3_	6	**3b**	80
3	4-F	-COCH_3_	5	**3c**	82
4	4-Cl	-COCH_3_	6	**3d**	78
5	4-Br	-COCH_3_	6	**3e**	56
6	4-CF_3_	-COCH_3_	6	**3f**	90
7	4-NO_2_	-COCH_3_	6	**3g**	81
8	5-F	-COOCH_3_	6	**3h**	77
9	4-Cl	-COOCH_3_	6	**3i**	72
10	3-F	-COOCH_3_	6	**3j**	78
11	H	-COOCH_2_CH_3_	6	**3k**	71
12	6-F	-COOCH_2_CH_3_	6	**3l**	73
13	5-F	-COOCH_2_CH_3_	6	**3m**	80
14	3-F	-COOCH_2_CH_3_	6	**3n**	81
15	4-Cl	-COOCH_2_CH_3_	6	**3o**	74
16	4-CF_3_	-COOCH_2_CH_3_	6	**3p**	90
17	4-NO_2_	-COOCH_2_CH_3_	6	**3q**	87

*^a^* Reaction conditions: 2-fluorobenzonitrile, **1** (1.0 equiv.); primary alcohols, **2** (1.0 equiv.); Cs_2_CO_3_ (3.0 equiv.). *^b^* Isolated yields.

**Table 3:** Synthesis of benzo[*b*]thiophen-3-amines *^a^.*

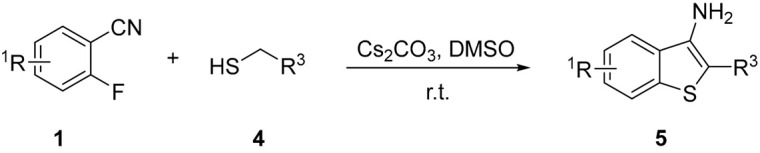					
**Entry**	**R^1^**	**R^3^**	**Time (h)**	**Product**	**Yield (%) *^b^***
1	3-F	-COCH_3_	6	**5a**	78
2	H	-COOCH_3_	6	**5b**	76
3	H	-COOCH_2_CH_3_	5	**5c**	74
4	H	-COOCH_2_CH_2_CH_2_CH_3_	6	**5d**	77
5	H	-ph	6	**5e**	85
6	5-F	-COCH_3_	6	**5f**	75
7	3-F	-COCH_3_	6	**5g**	76
8	5-F	-COOCH_3_	6	**5h**	75
9	4-OMe	-COOCH_2_CH_3_	6	**5i**	31

*^a^* Reaction conditions: 2-fluorobenzonitrile, **1** (1.0 equiv.); α-substituted methanethiol, **4** (1.0 equiv.); Cs_2_CO_3_ (3.0 equiv.). *^b^* Isolated yields.

## Data Availability

The data used to support the findings of this study are available from the corresponding author upon request.

## Supplementary Materials

The following supporting information can be downloaded at: https://www.mdpi.com/article/10.3390/molecules28237738/s1, Table S1: Details of Crystal Structure Determination for **5g**; Figure S1: X-ray structure of compound **5g**; Figure S2: ^1^H NMR and ^13^C NMR spectra of compound **3a**; Figure S3: ^1^H NMR and ^13^C NMR spectra of compound **3b**; Figure S4: ^1^H NMR and ^13^C NMR spectra of compound **3c**; Figure S5: ^1^H NMR and ^13^C NMR spectra of compound **3d**; Figure S6: ^1^H NMR and ^13^C NMR spectra of compound **3e**; Figure S7: ^1^H NMR and ^13^C NMR spectra of compound **3f**; Figure S8: ^1^H NMR and ^13^C NMR spectra of compound **3g**; Figure S9: ^1^H NMR and ^13^C NMR spectra of compound **3h**; Figure S10: ^1^H NMR and ^13^C NMR spectra of compound **3i**; Figure S11: ^1^H NMR and ^13^C NMR spectra of compound **3j**; Figure S12: ^1^H NMR and ^13^C NMR spectra of compound **3k**; Figure S13: ^1^H NMR and ^13^C NMR spectra of compound **3l**; Figure S14: ^1^H NMR and ^13^C NMR spectra of compound **3m**; Figure S15: ^1^H NMR and ^13^C NMR spectra of compound **3n**; Figure S16: ^1^H NMR and ^13^C NMR spectra of compound **3o**; Figure S17: ^1^H NMR and ^13^C NMR spectra of compound **3p**; Figure S18: ^1^H NMR and ^13^C NMR spectra of compound **3q**; Figure S19: ^1^H NMR and ^13^C NMR spectra of compound **5a**; Figure S20: ^1^H NMR and ^13^C NMR spectra of compound **5b**; Figure S21: ^1^H NMR and ^13^C NMR spectra of compound **5c**; Figure S22: ^1^H NMR and ^13^C NMR spectra of compound **5d**; Figure S23: ^1^H NMR and ^13^C NMR spectra of compound **5e**; Figure S24: ^1^H NMR and ^13^C NMR spectra of compound **5f**; Figure S25: ^1^H NMR and ^13^C NMR spectra of compound **5g**; Figure S26: ^1^H NMR and ^13^C NMR spectra of compound **5h**; Figure S27: ^1^H NMR and ^13^C NMR spectra of compound **5i**.

## References

[1] Nevagi R.J., Dighe S.N., Dighe S.N. (2015). Biological and medicinal significance of benzofuran. Eur. J. Med. Chem..

[2] Keri R.S., Chand K., Budagumpi S., Somappa S.B., Patil S.A., Nagaraja B.A. (2017). An overview of benzo [*b*] thiophene-based medicinal chemistry. Eur. J. Med. Chem..

[3] Khanam H., Shamsuzzaman (2015). Bioactive Benzofuran derivatives: A review. Eur. J. Med. Chem..

[4] Miao Y., Hu Y., Yang J., Liu T., Sun J., Wang X. (2019). Natural source, bioactivity and synthesis of benzofuran derivatives. RSC Adv..

[5] de Brito D.H.A., Almeida-Neto F.W.Q., Ribeiro L.R. (2022). Synthesis, structural and spectroscopic analysis, and antiproliferative activity of chalcone derivate (E)-1-(4-aminophenyl)-3-(benzo [*b*] thiophen-2-yl) prop-2-en-1-one in Trypanosoma cruzi. J. Mol. Struct..

[6] Algso M.A.S., Kivrak A. (2019). New strategy for the synthesis of 3-ethynyl-2-(thiophen-2-yl) benzo [*b*] thiophene derivatives. Chem. Pap..

[7] Dhillon S., Clarke M. (2014). Tasimelteon: First global approval. Drugs.

[8] Mhoumadi A., Elkhashab M., Prillieux S., Dumas J., Collas F., Louvain N., Fraisse B., Espeau P. (2022). Characterization of the heat behavior of amiodarone hydrochloride. Thermochim. Acta.

[9] McGovern B., Garan H., Kelly E., Ruskin J.N. (1983). Adverse reactions during treatment with amiodarone hydrochloride. Br. Med. J. (Clin. Res. Ed.).

[10] Shirley M. (2018). Fruquintinib: First global approval. Drugs.

[11] Li J., Qin S., Xu R.H. (2018). Effect of fruquintinib vs placebo on overall survival in patients with previously treated metastatic colorectal cancer: The FRESCO randomized clinical trial. JAMA.

[12] Valkova S., Trashlieva M., Christova P. (2004). Treatment of vitiligo with local khellin and UVA: Comparison with systemic PUVA. Clin. Exp. Dermatol..

[13] Berrade L., Aisa B., Ramirez M.J., Galiano S., Guccione S., Moltzau L.R., Levy F.O., Nicoletti F., Battaglia G., Molinaro G. (2011). Novel Benzo[*b*]thiophene Derivatives as New Potential Antidepressants with Rapid Onset of Action. J. Med. Chem..

[14] Liu Z., Xia Y., Zhou S., Wang L., Zhang Y., Wang J. (2013). Pd-catalyzed cyclization and carbene migratory insertion: New approach to 3-vinylindoles and 3-vinylbenzofurans. Org. Lett..

[15] Zhang P., Wang C., Cui M., Du M., Li W., Jia Z., Zhao Q. (2020). Synthesis of difluoroalkylated benzofuran, benzothiophene, and indole derivatives via palladium-catalyzed cascade difluoroalkylation and arylation of 1, 6-enynes. Org. Lett..

[16] Liao J., Fan L., Guo W., Zhang Z., Li J., Zhu C., Ren Y., Wu W., Jiang H. (2017). Palladium-Catalyzed Fluoroalkylative Cyclization of Olefins. Org. Lett..

[17] Kuram M.R., Bhanuchandra M., Sahoo A.K. (2013). Direct access to benzo [*b*] furans through Palladium-catalyzed oxidative annulation of phenols and unactivated internal alkynes. Angew. Chem. Int. Ed..

[18] Huang X., Rong N., Li P., Shen G., Li Q., Xin N., Cui C., Cui J., Yang B., Li D. (2018). AIBN-promoted synthesis of bibenzo [*b*][1, 4] thiazines by the condensation of 2, 2′-dithiodianiline with methyl aryl ketones. Org. Lett..

[19] Wang D., Lu Q., Li Z., Fang C., Liu R., Yang B., Shen G. (2022). “One-Pot” CuCl_2_-Mediated Condensation/C–S Bond Coupling Reactions to Synthesize Dibenzothiazepines by Bi-Functional-Reagent N, N′-Dimethylethane-1, 2-Diamine. Molecules.

[20] Liu G., Liu S., Li Z., Chen C., Li J., Zhang Y., Shen G., Yang B., Hu X., Huang X. (2022). Metal-and oxidant-free electrochemically promoted oxidative coupling of amines. RSC Adv..

[21] Khan I., Zaib S., Ibrar A. (2020). New frontiers in the transition-metal-free synthesis of heterocycles from alkynoates: An overview and current status. Org. Chem. Front..

[22] Suleymanov A.A., Scopelliti R., Fadaei Tirani F., Severin K. (2018). One-Pot Synthesis of Trisubstituted Triazenes from Grignard Reagents and Organic Azides. Org. Lett..

[23] Li Y., Zhan C., Yang B., Cao X., Ma C. (2013). A one-pot transition-metal-free tandem process to 1, 4-benzodiazepine scaffolds. Synthesis.

[24] Crystal Data for **5g**: See the Supporting Information. CCDC 2285416 (**5g**) Contains the Supplementary Crystallographic Data for This Paper. These Data Can Be Obtained Free of Charge from The Cambridge Crysallographic Data Centre. www.ccdc.cam.ac.uk/data_request/cif.

[25] Sheldrick G.M. (1997). SHELX-97. Program for the Refinement of Crystal Structure.

