# Supplementary material for: Transition-Metal-Free One-Pot Synthesis of Fused Benzofuranamines and Benzo[b]thiophenamines
Source: Molecules. 2023 Nov 23;28(23):7738. doi: 10.3390/molecules28237738 (PMC10708444; doi:10.3390/molecules28237738)
Supplement: Supplementary file 1 [file molecules-28-07738-s001.zip › molecules-2695468-supplementary.pdf]

Transition Metal-Free One-Pot Synthesis of Fused Benzofuranamines and Benzo[*b*]thiophenamines

Ran Liu[1], Lili Lv[2], Bingchuan Yang*[1,3,4,5], Ziyi Gu[1], Chenglong Li [6], Xueyan Lv[4], Chengcheng Ding[4], Xianqiang Huang[1], Dong Yuan*[3]

[1] School of Chemistry and Chemical Engineering, Liaocheng University, Liaocheng 252000, China;

[2] China Petroleum Planning and Engineering Institute, Dongying 257237, China;

[3] College of Chemistry and Chemical Engineering, Qilu Normal University, Jinan, 250013, China;.

[4] School of Chemistry and Chemical Engineering, Shandong University, Jinan 250100, China.

[5] The Department of Chemistry, University of South Florida, 4202 East Fowler Avenue, Tampa, Florida 33620, United States

[6] Shandong Weijiao Holding Group Co., Ltd, Weifang, 262404, China.

Table of contents

1. X-ray data and structure for product 5g..3

2. Copies of ^{1}H NMR and ^{13}C NMR spectra of 3a-3q, 5a-5i..........4-25

1. X-ray data and structure for product 5g

Table S1. Details of Crystal Structure Determination for **5g**.

Compound	**5g**
Chemical formula	$C_{10}H_8FNO_2S$
Formula Mass	225.03
Crystal system	monoclinic
a/Å	13.7929(8)
b/Å	3.9422(3)
c/Å	22.5228(15)
α/°	90.00
β/°	125.910(5)
γ/°	90.00
Unit cell volume/Å^3	991.90(12)
Temperature/K	293
Space group	$P21/c$
No. of formula units per unit cell, Z	4
No. of reflections measured	1137
No. of independent reflections	1781
R_{int}	0.0406
Final R_1 values ($I > 2\sigma(I)$)	0.0637
Final $wR(F^2)$ values ($I > 2\sigma(I)$)	0.1604
Final R_1 values (all data)	0.0997
Final $wR(F^2)$ values (all data)	0.2019

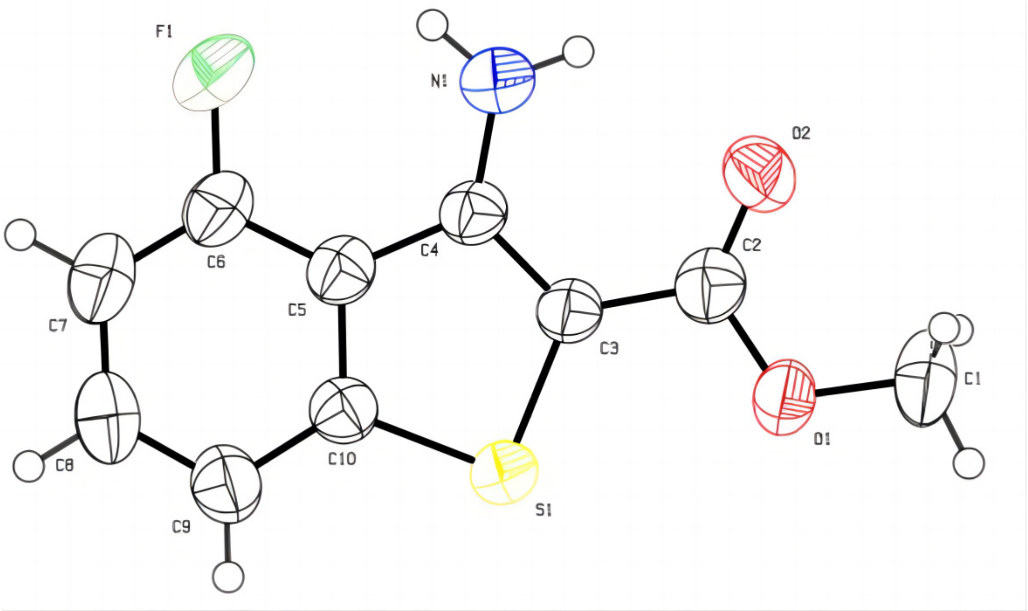

Figure S1. X-ray structure of compound **5g**

2. Copies of ^{1}H NMR and ^{13}C NMR spectra of 3a-3q, 5a-5i

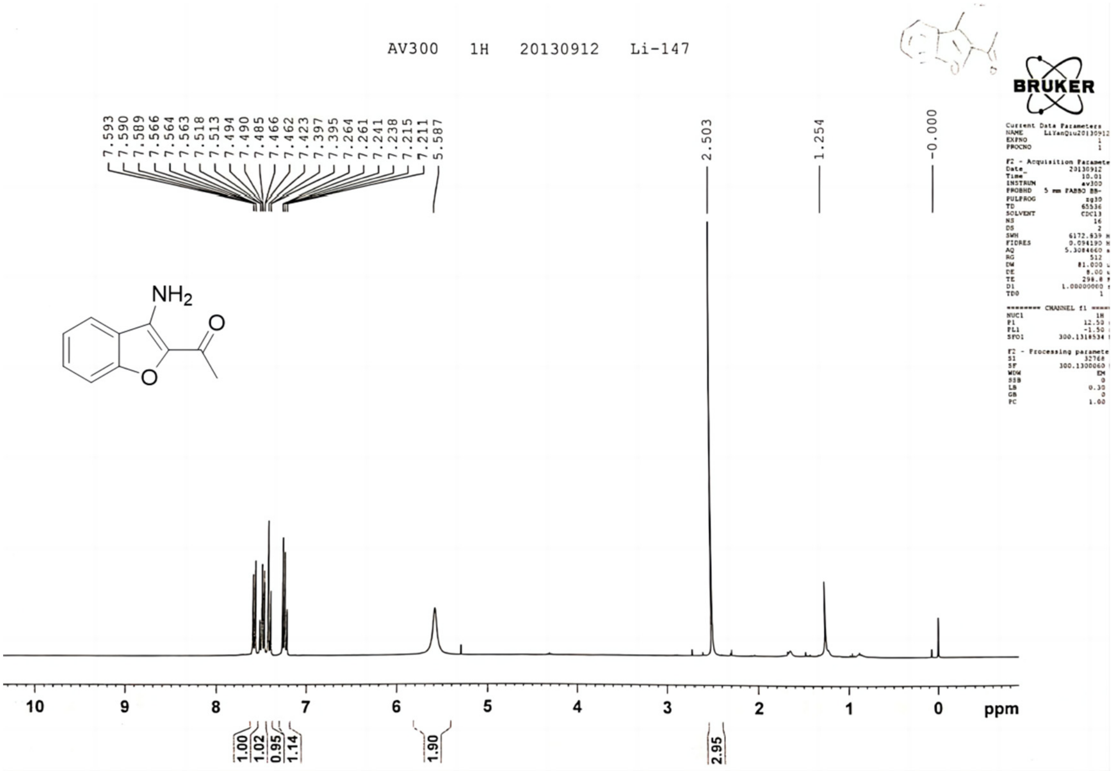

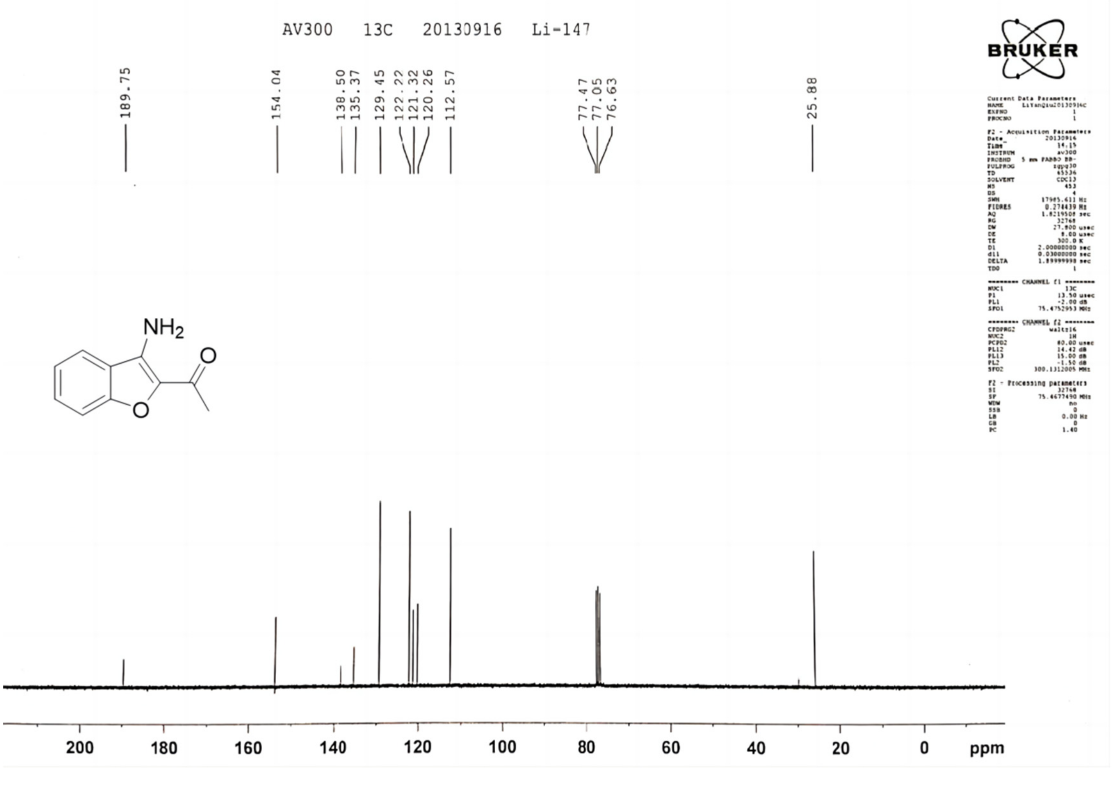

Figure S2. ^{1}H NMR and ^{13}C NMR spectra of compound **3a**

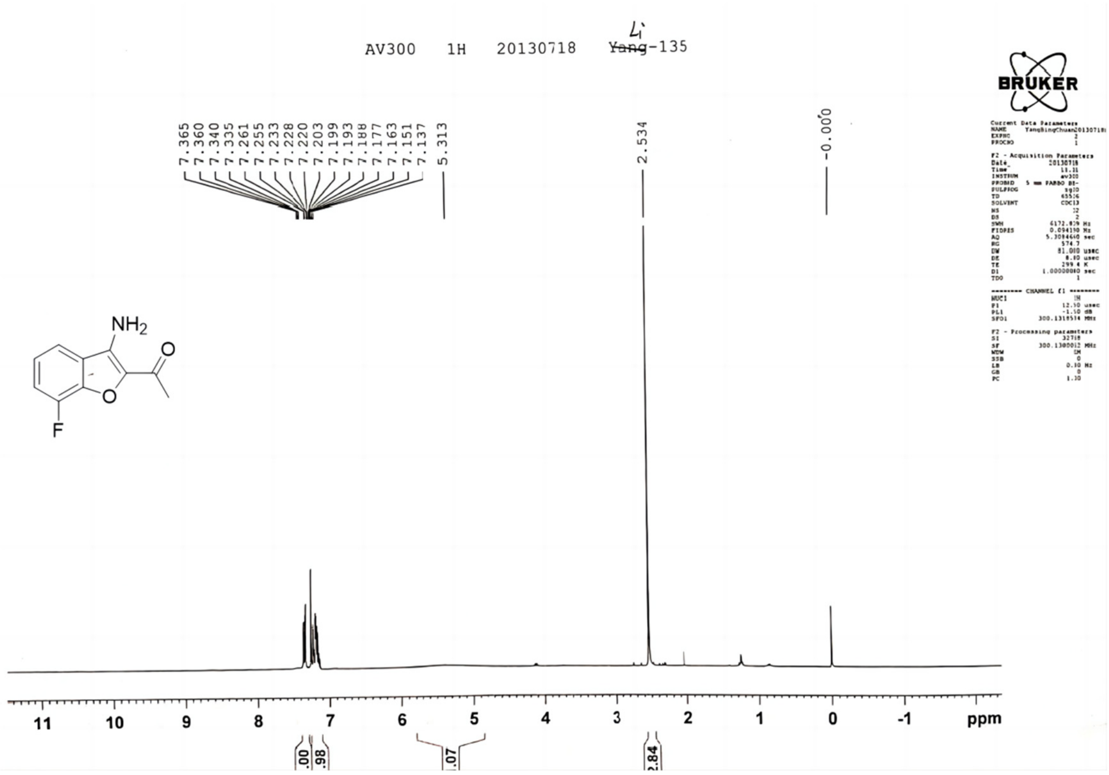

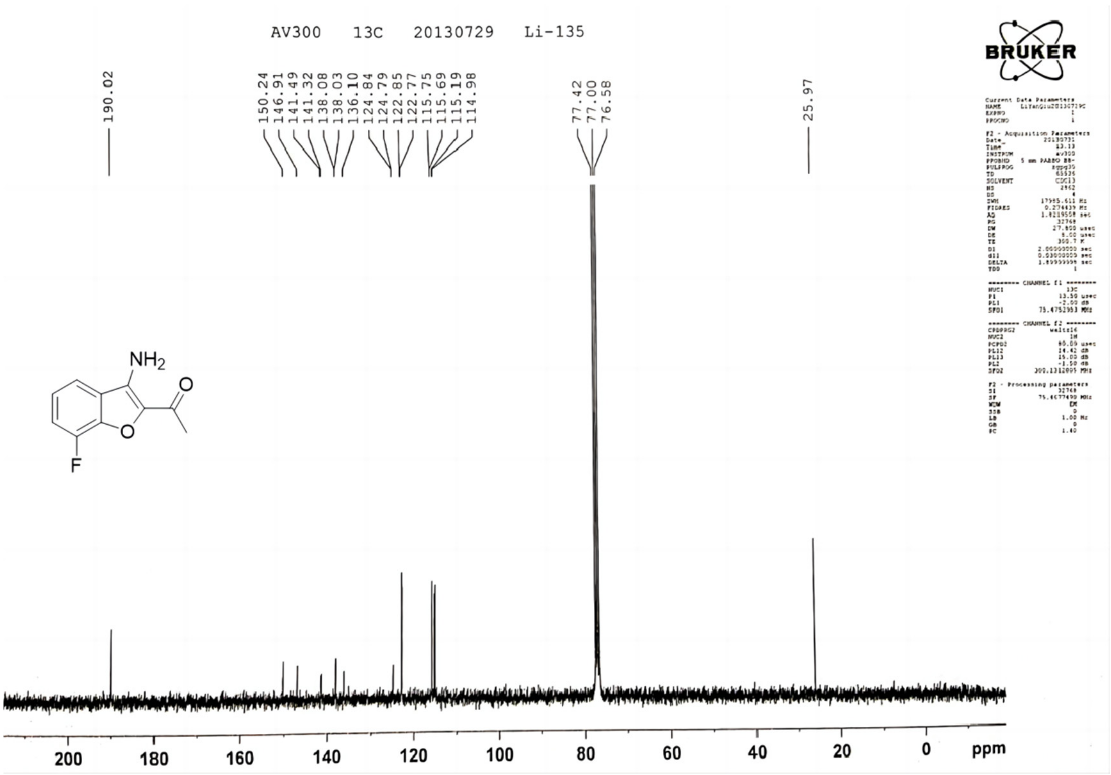

Figure S3. ^{1}H NMR and ^{13}C NMR spectra of compound **3b**

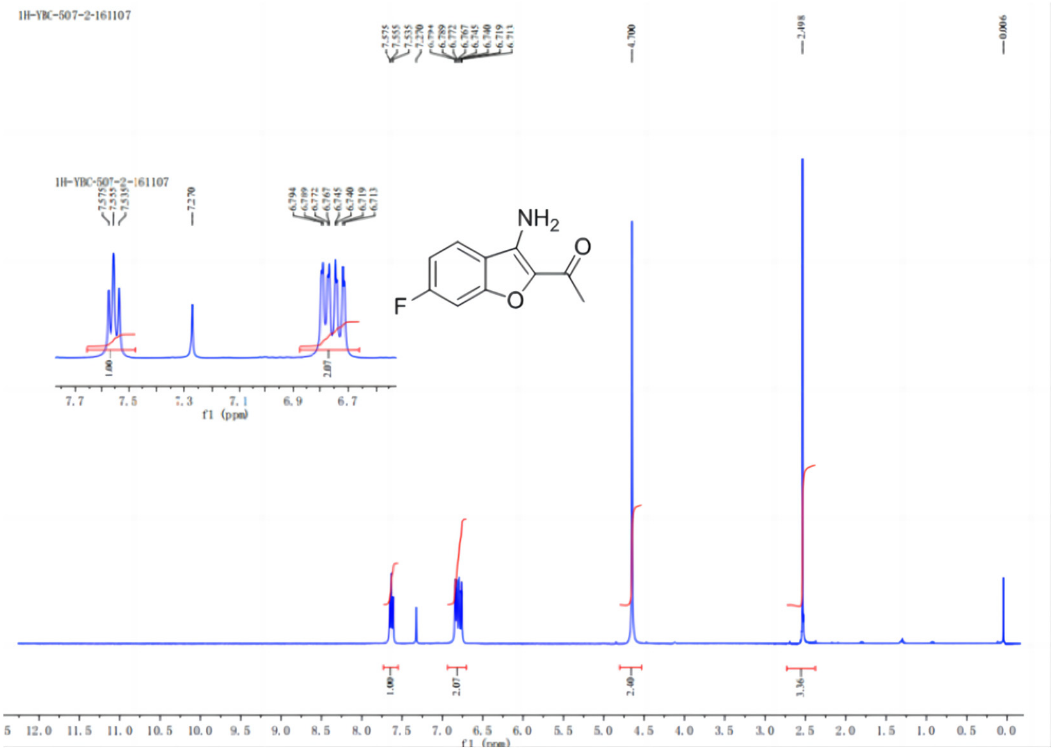

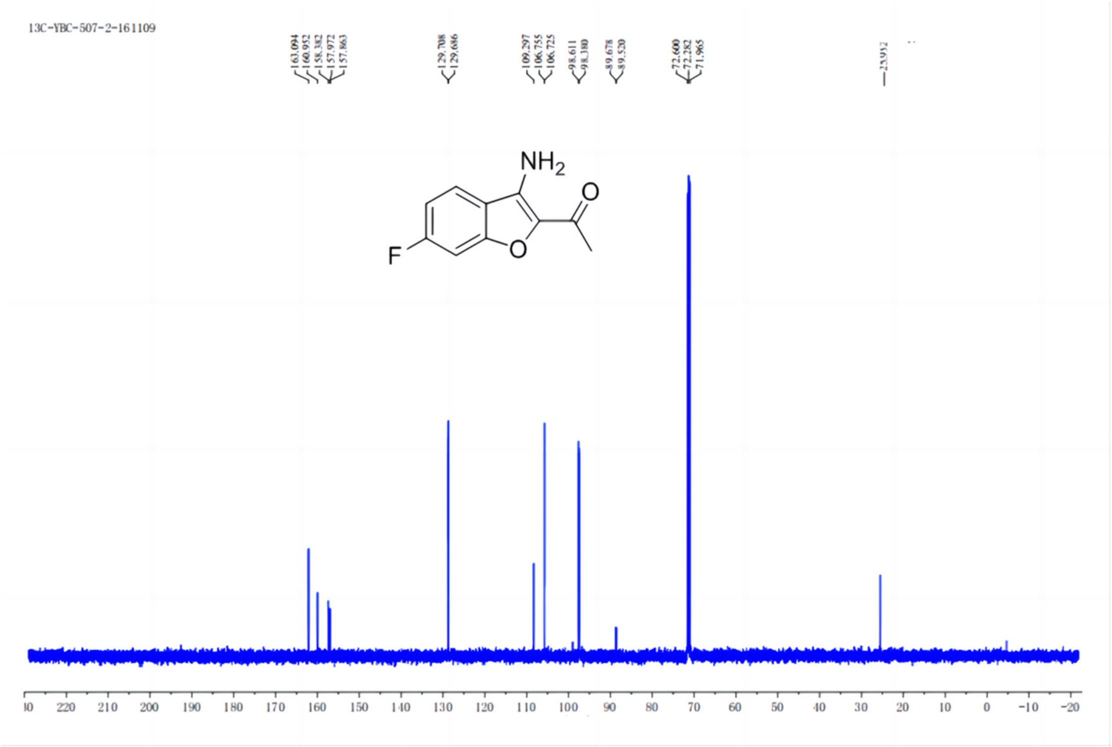

Figure S4. ^{1}H NMR and ^{13}C NMR spectra of compound **3c**

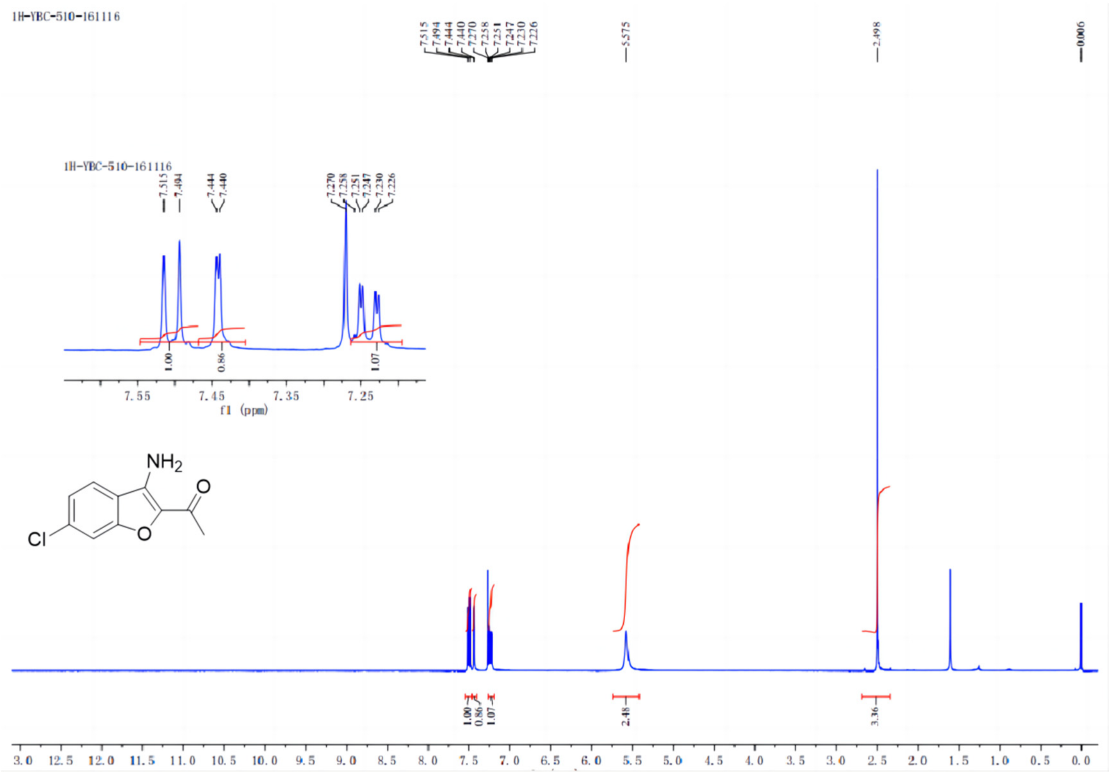

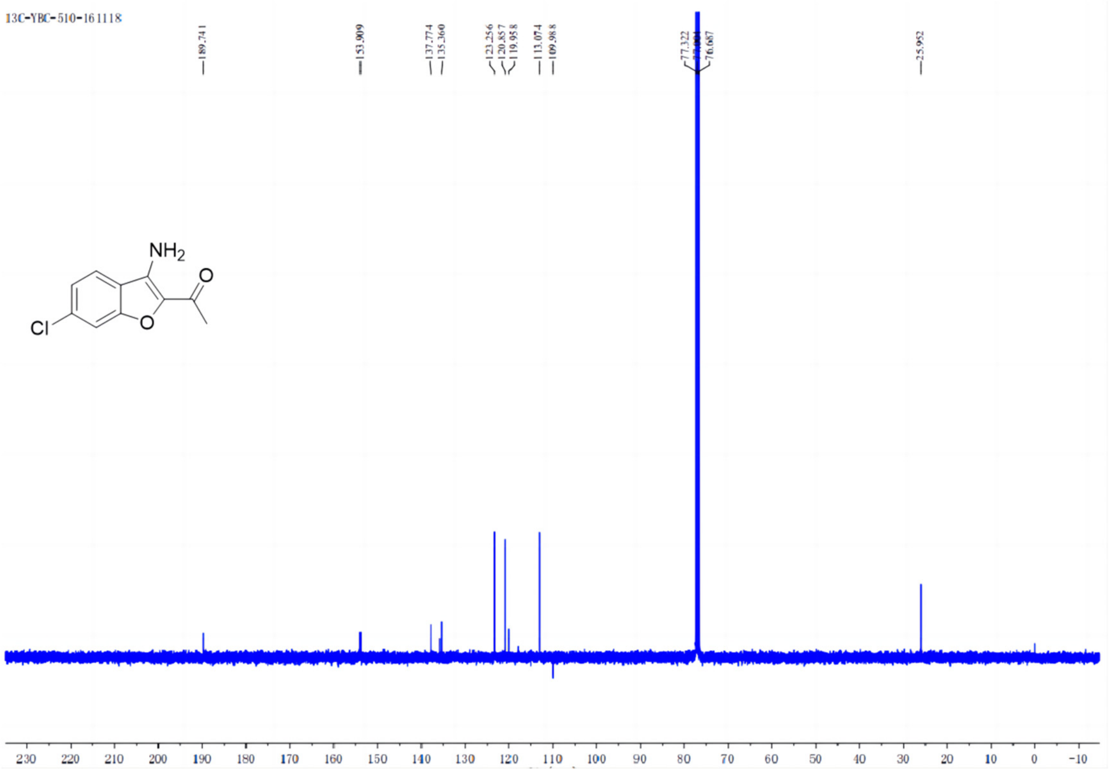

Figure S5. ^{1}H NMR and ^{13}C NMR spectra of compound **3d**

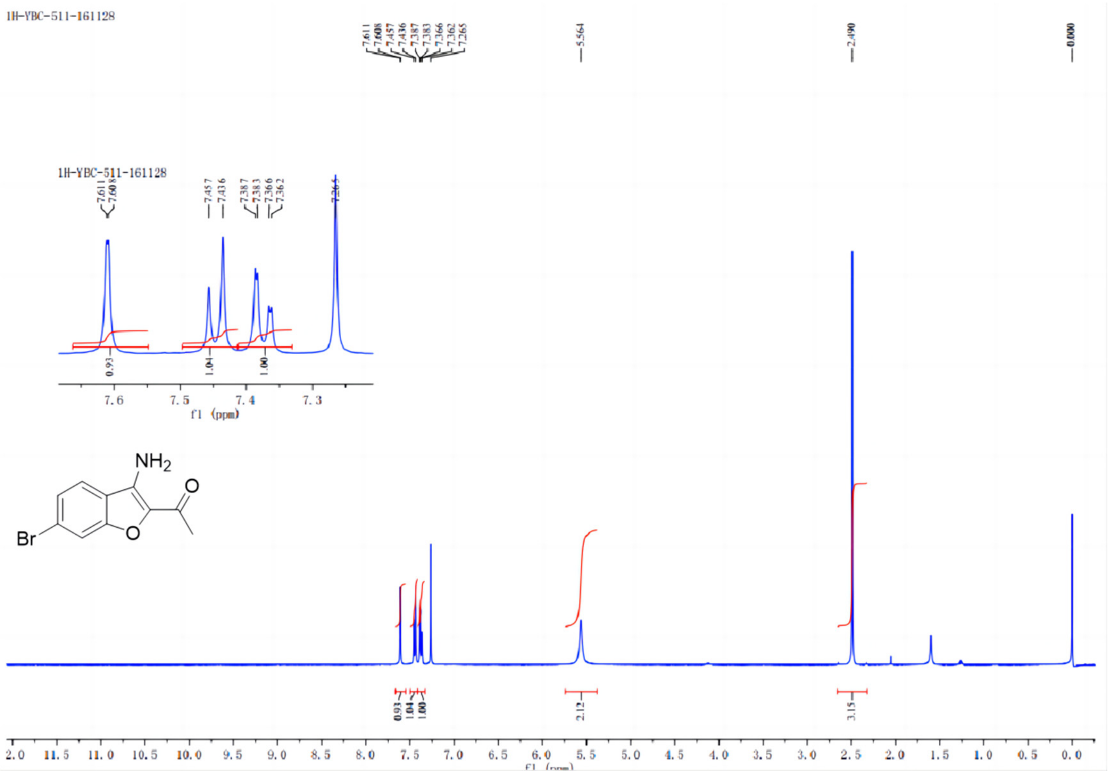

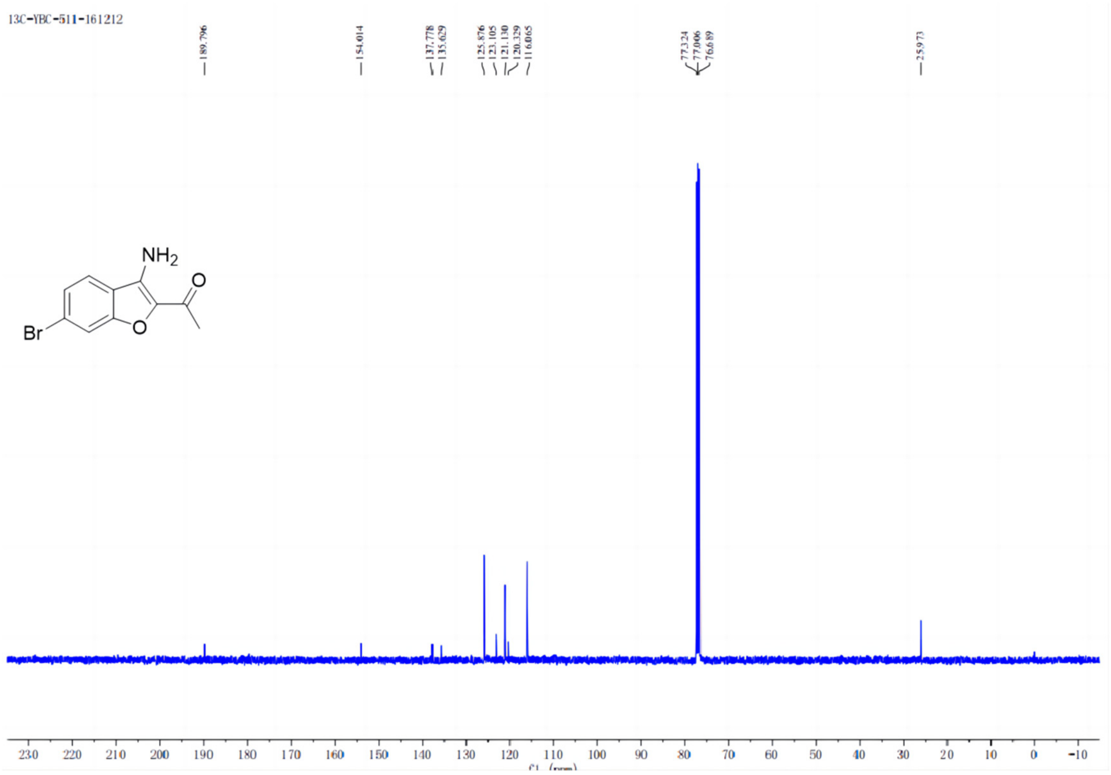

Figure S6. ^{1}H NMR and ^{13}C NMR spectra of compound **3e**

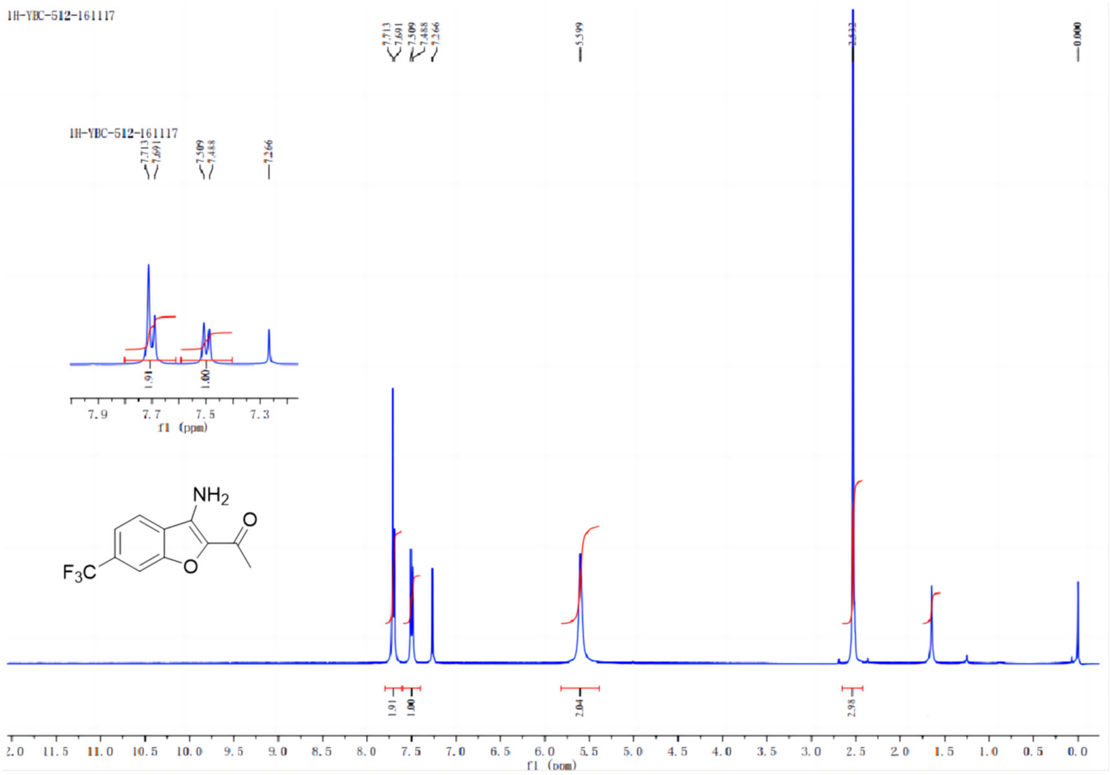

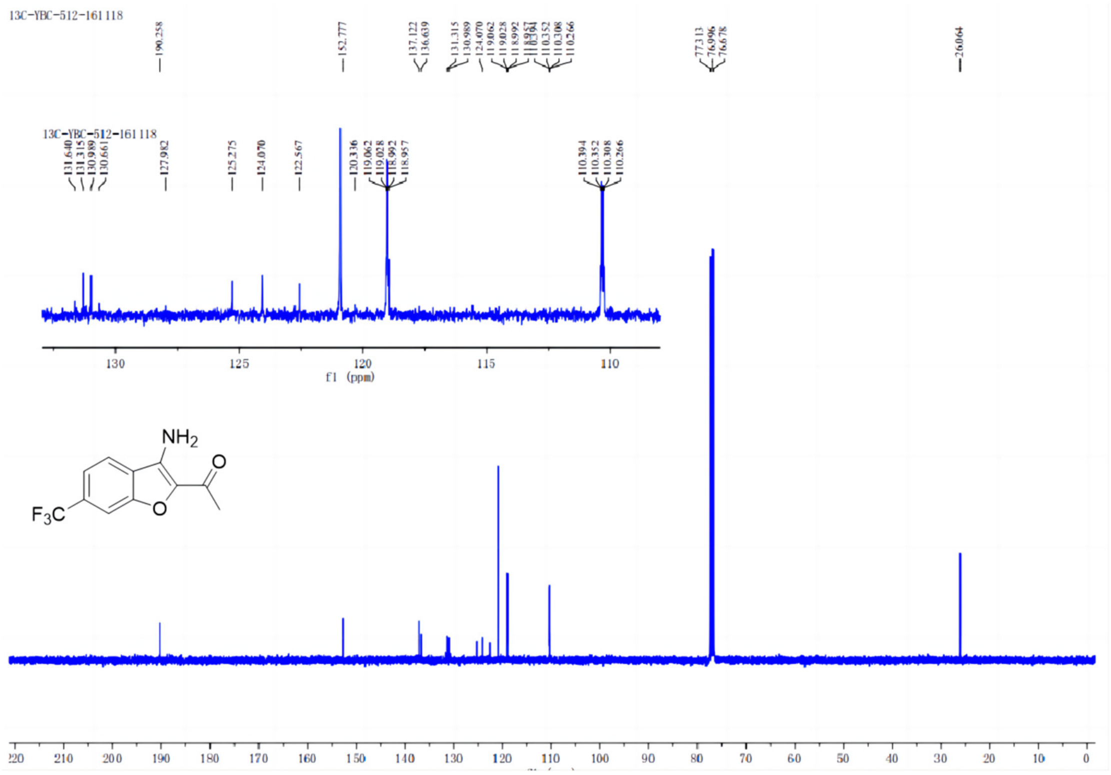

Figure S7. ^{1}H NMR and ^{13}C NMR spectra of compound **3f**

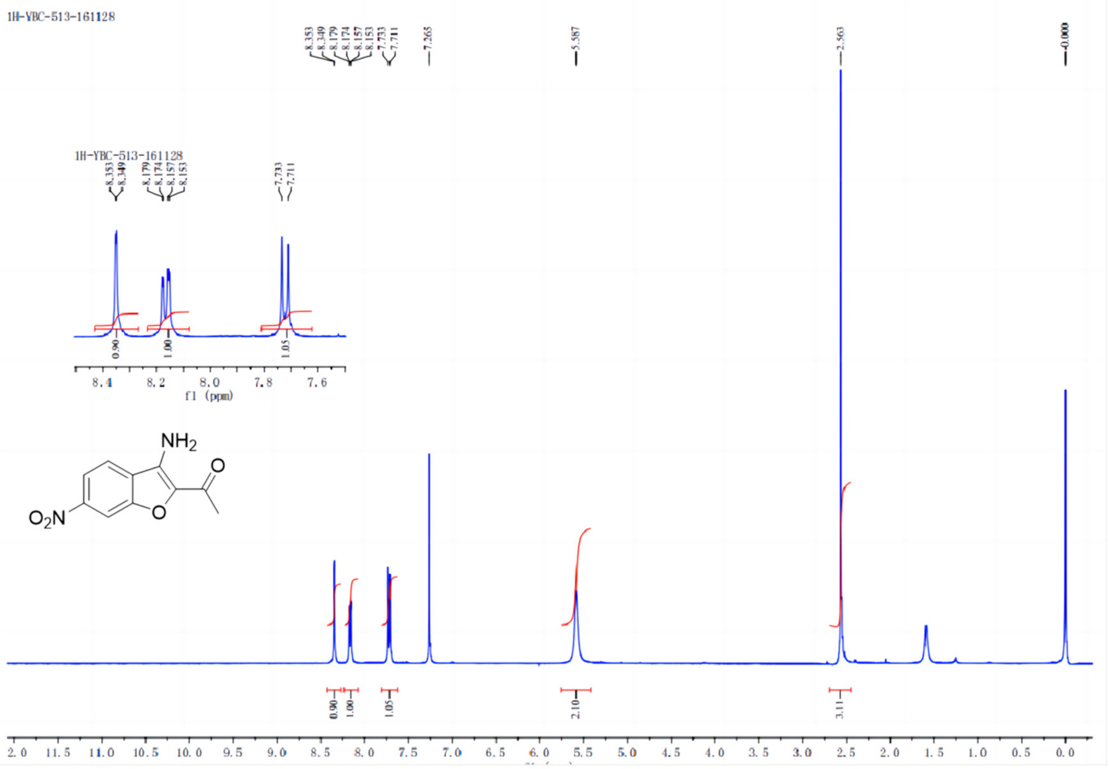

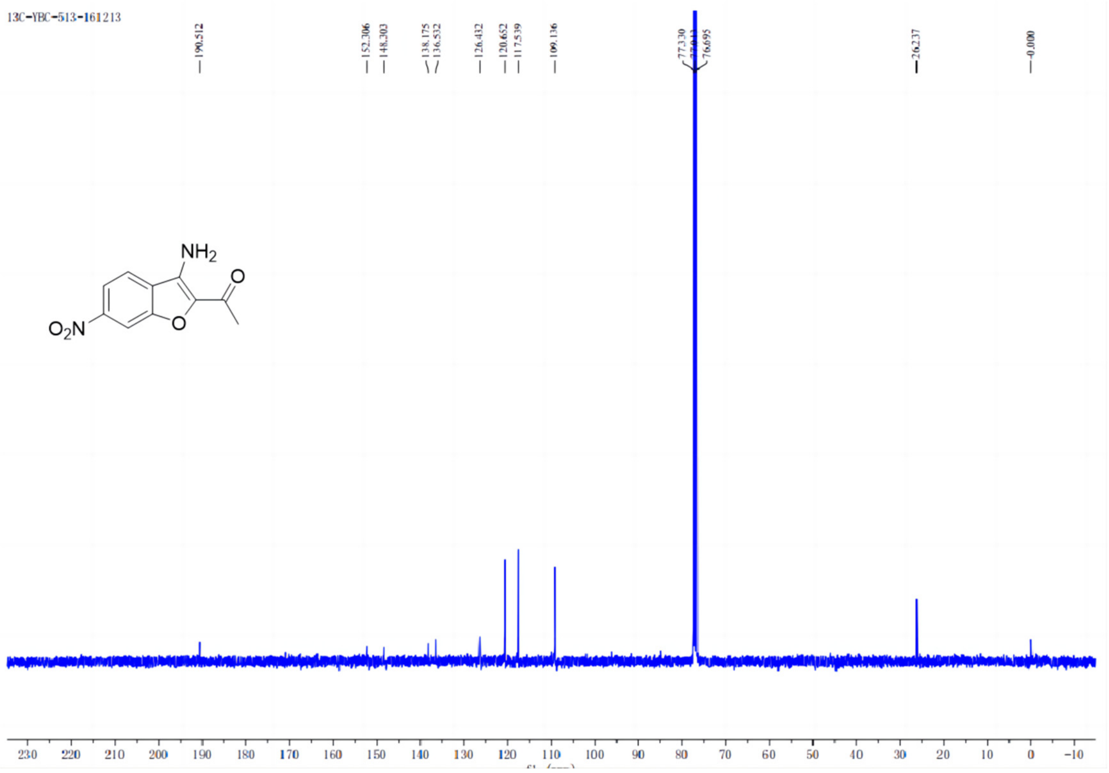

Figure S8. ^{1}H NMR and ^{13}C NMR spectra of compound **3g**

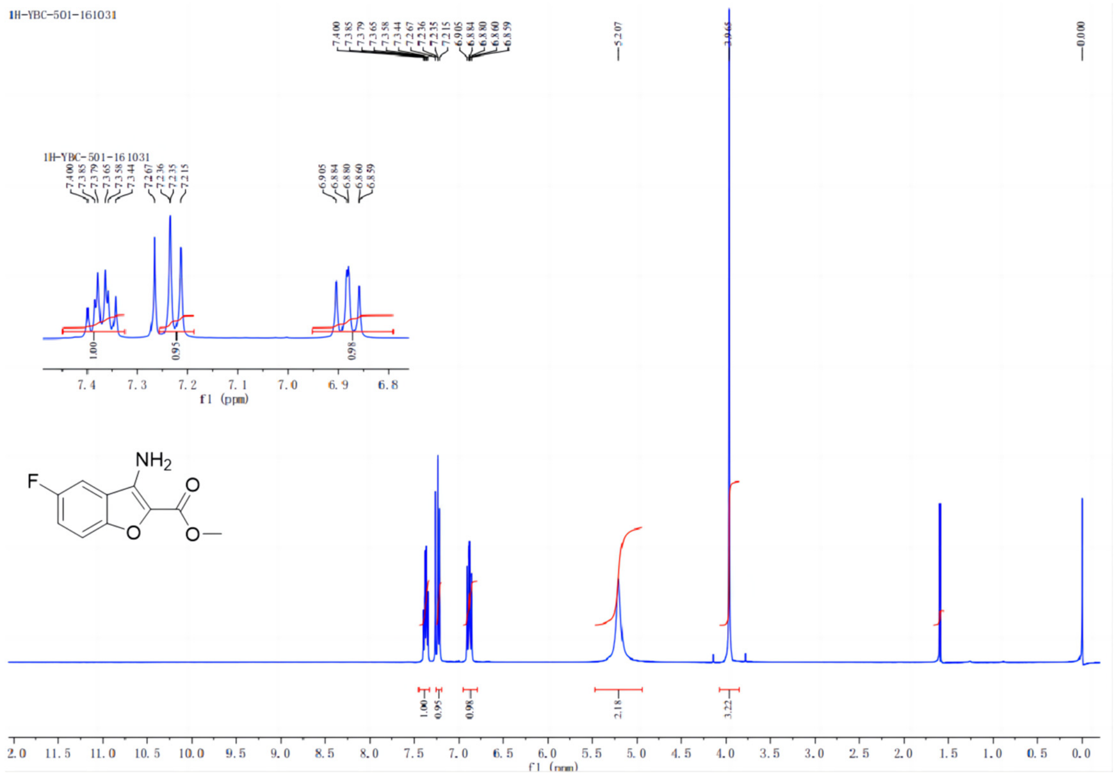

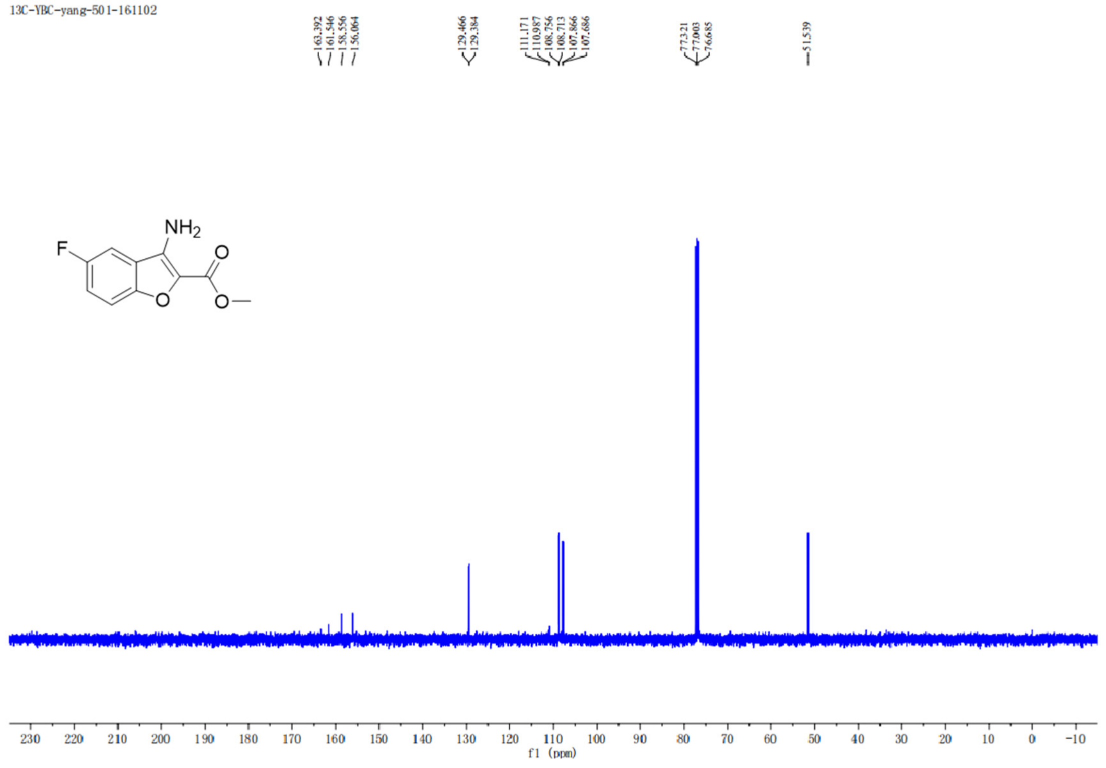

Figure S9. ^{1}H NMR and ^{13}C NMR spectra of compound **3h**

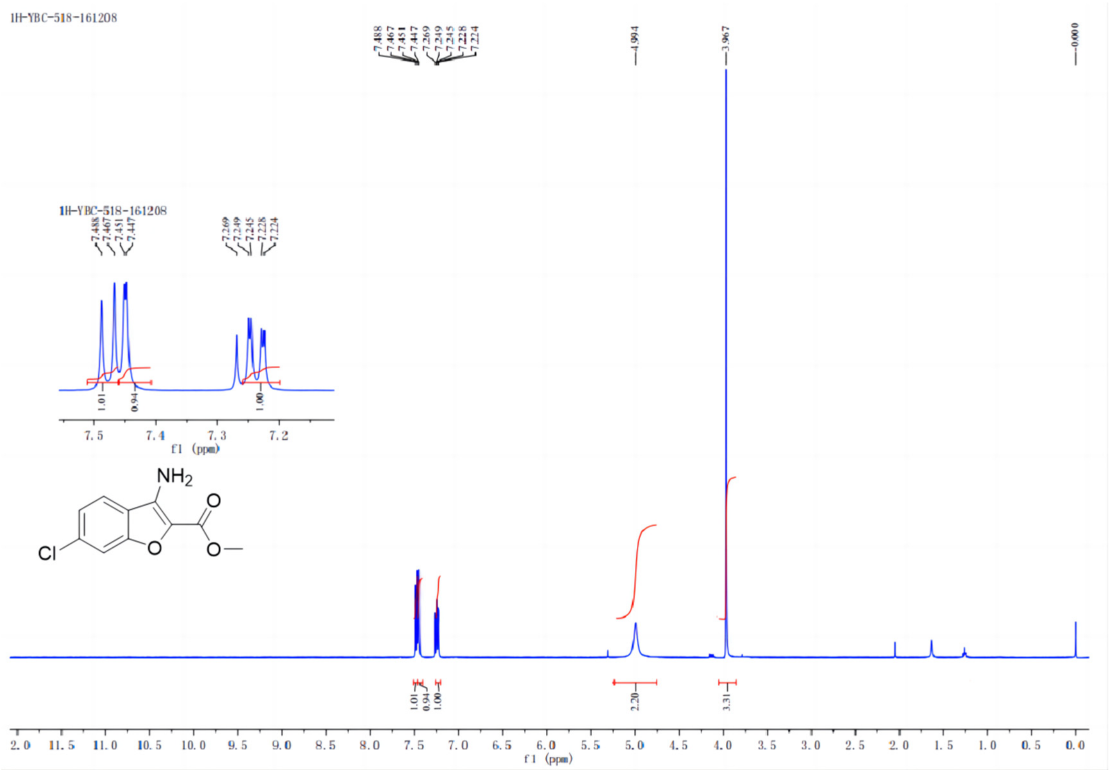

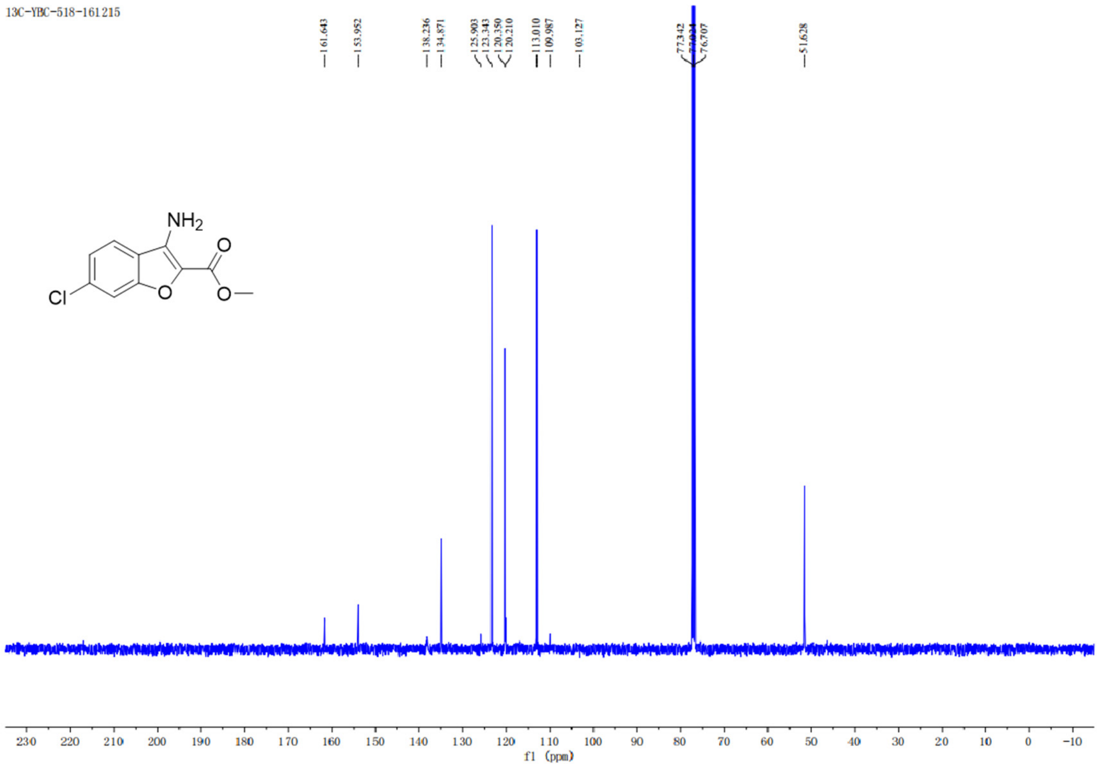

Figure S10. ^{1}H NMR and ^{13}C NMR spectra of compound **3i**

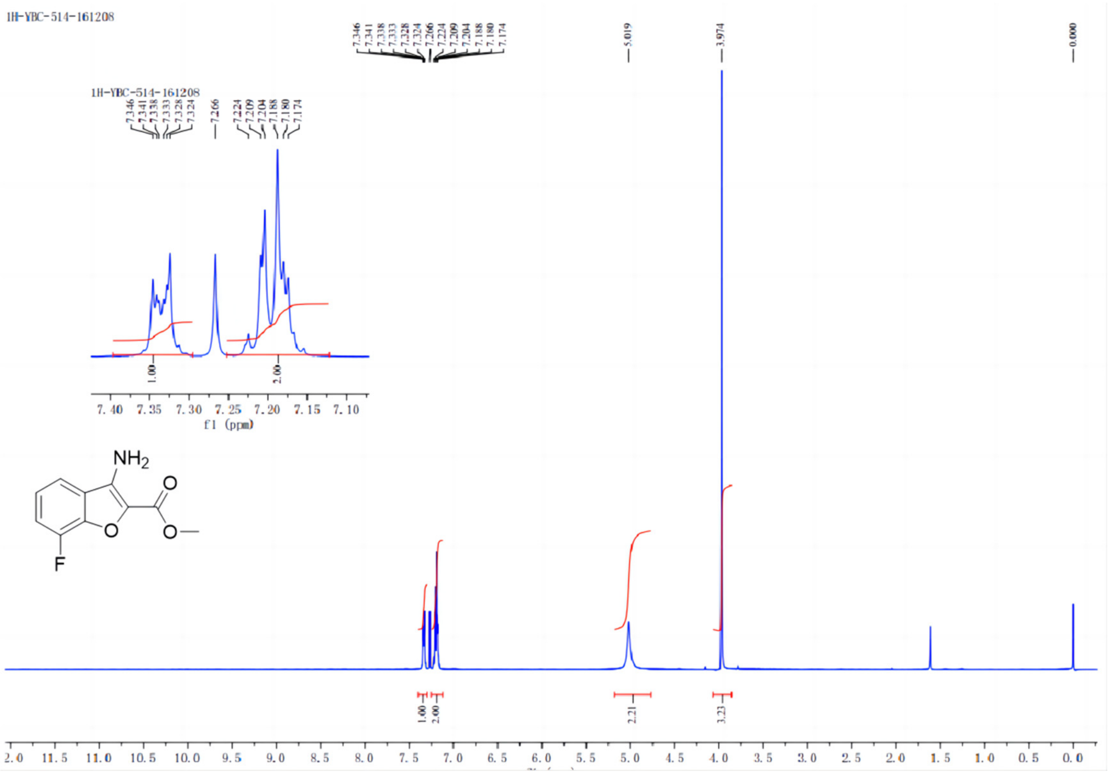

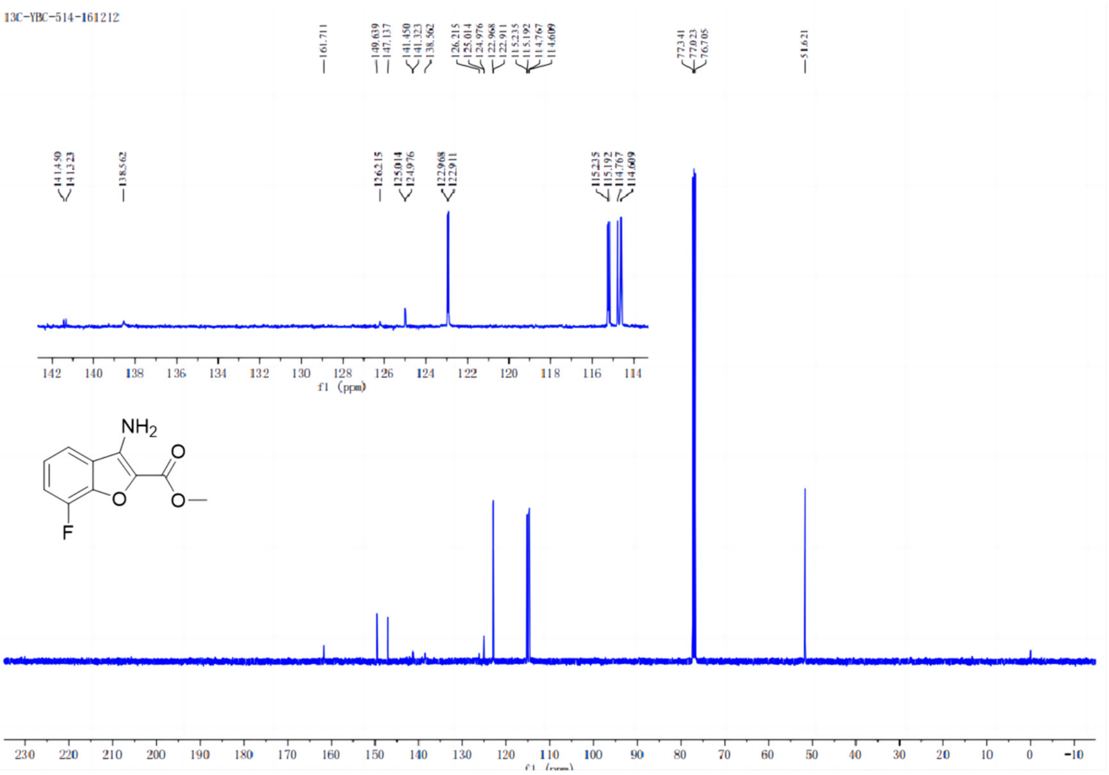

Figure S11. ^{1}H NMR and ^{13}C NMR spectra of compound **3j**

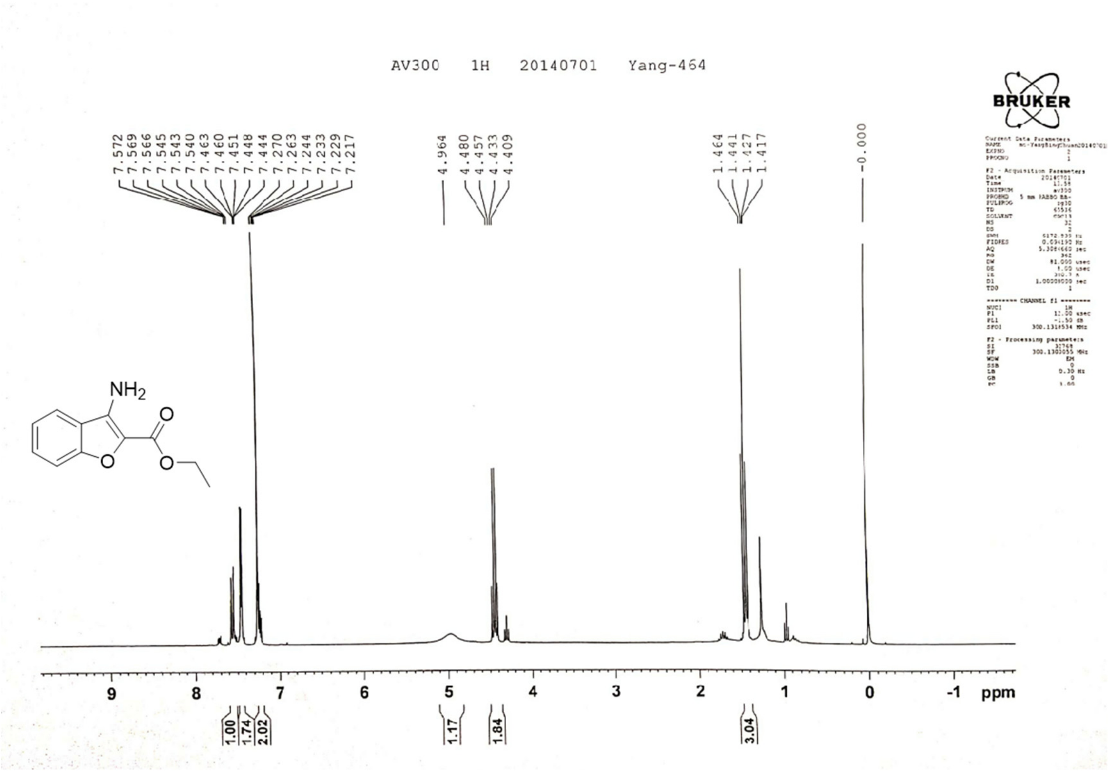

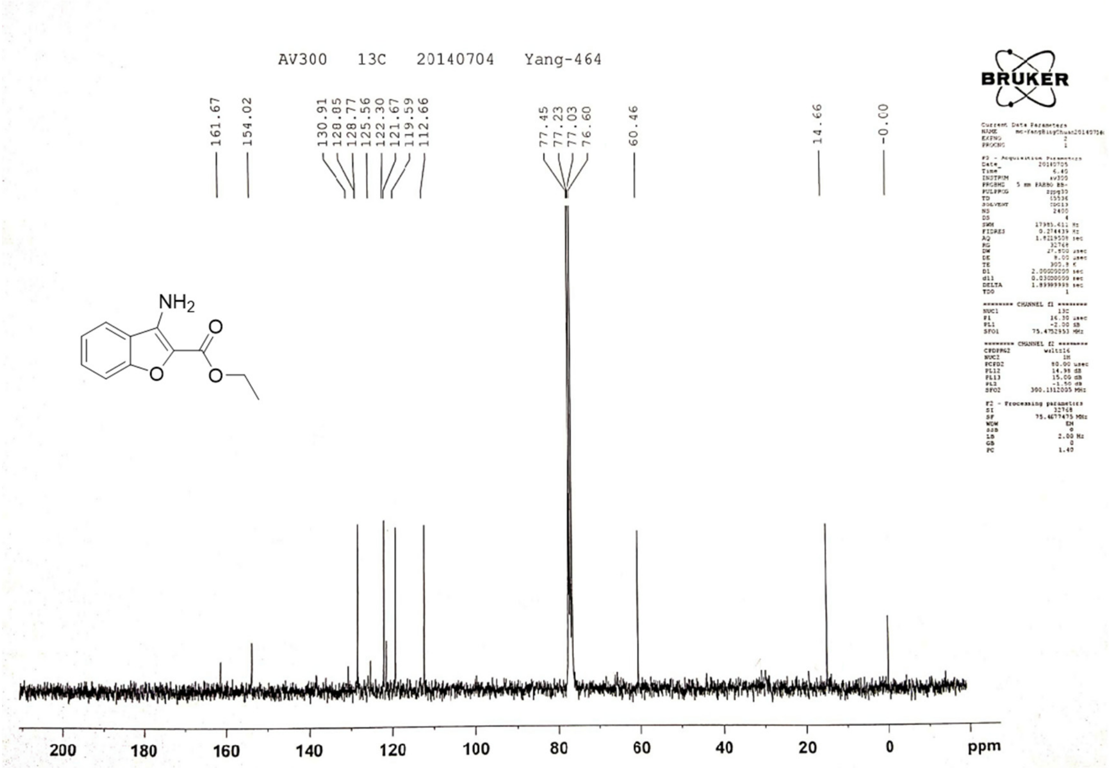

Figure S12. ^{1}H NMR and ^{13}C NMR spectra of compound **3k**

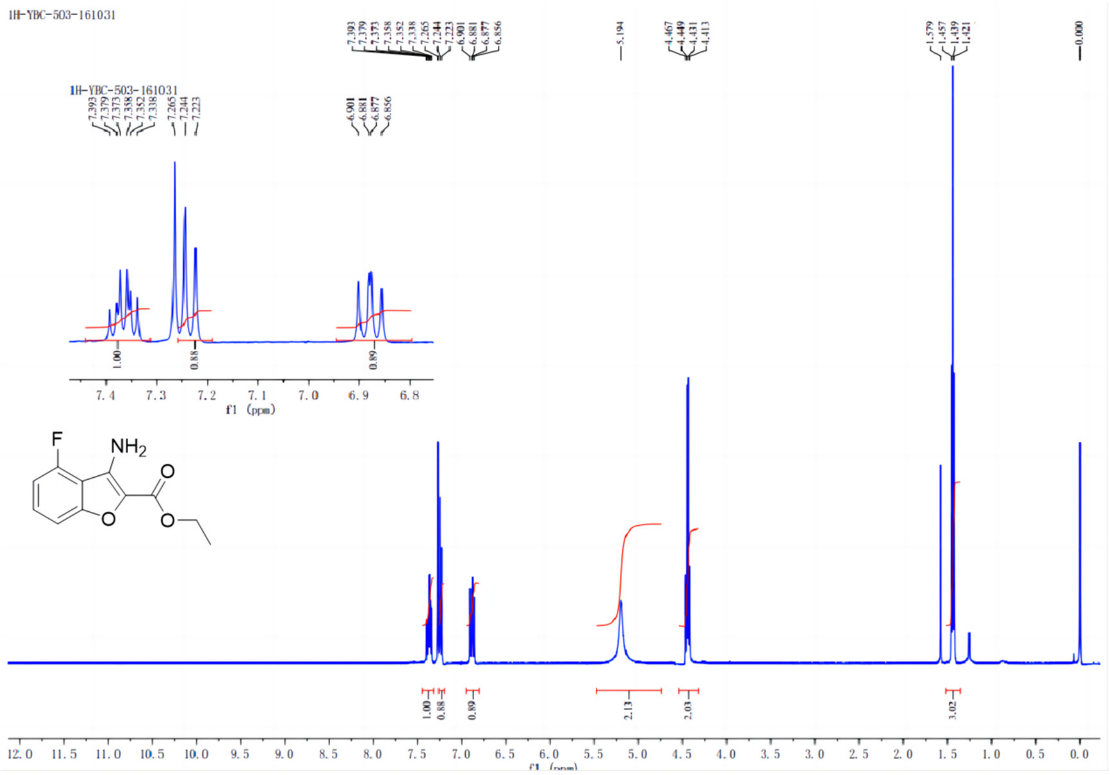

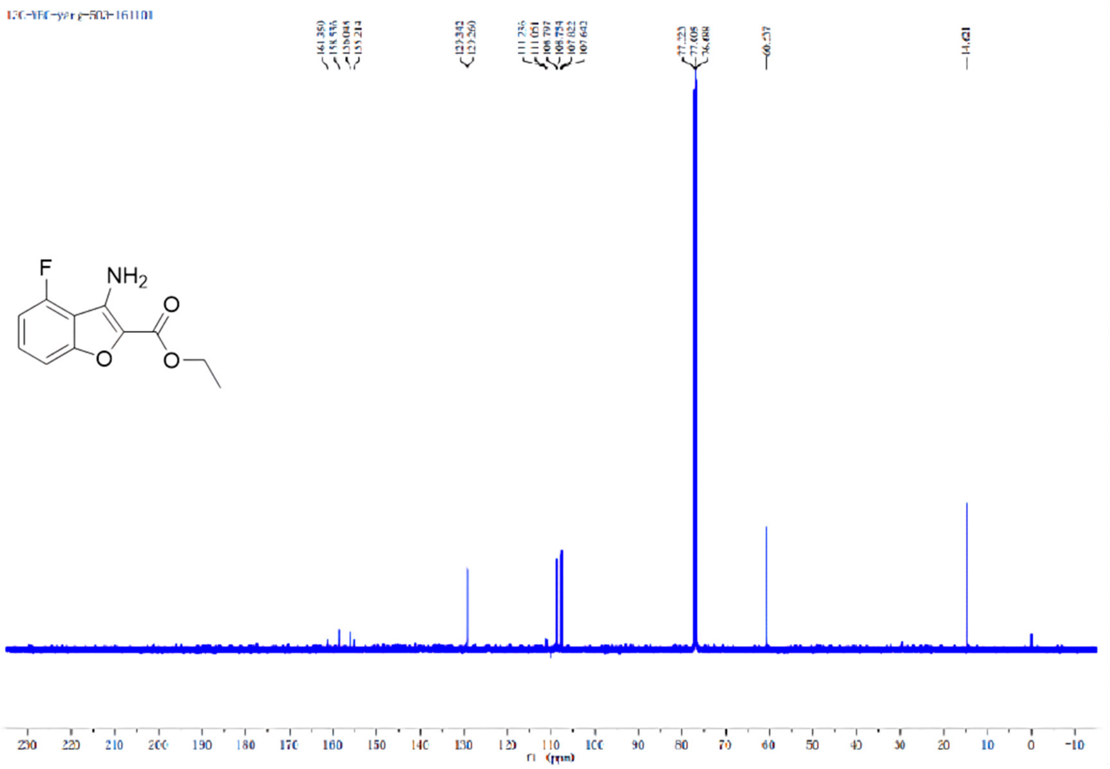

Figure S13. ^{1}H NMR and ^{13}C NMR spectra of compound **3l**

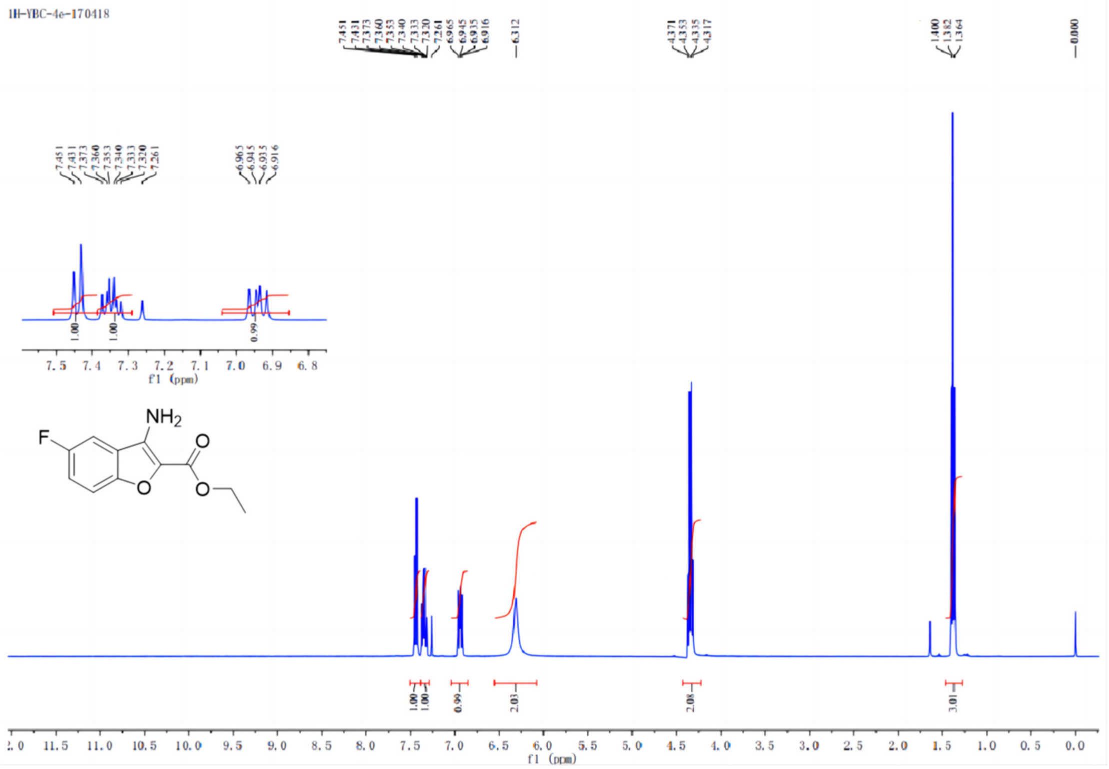

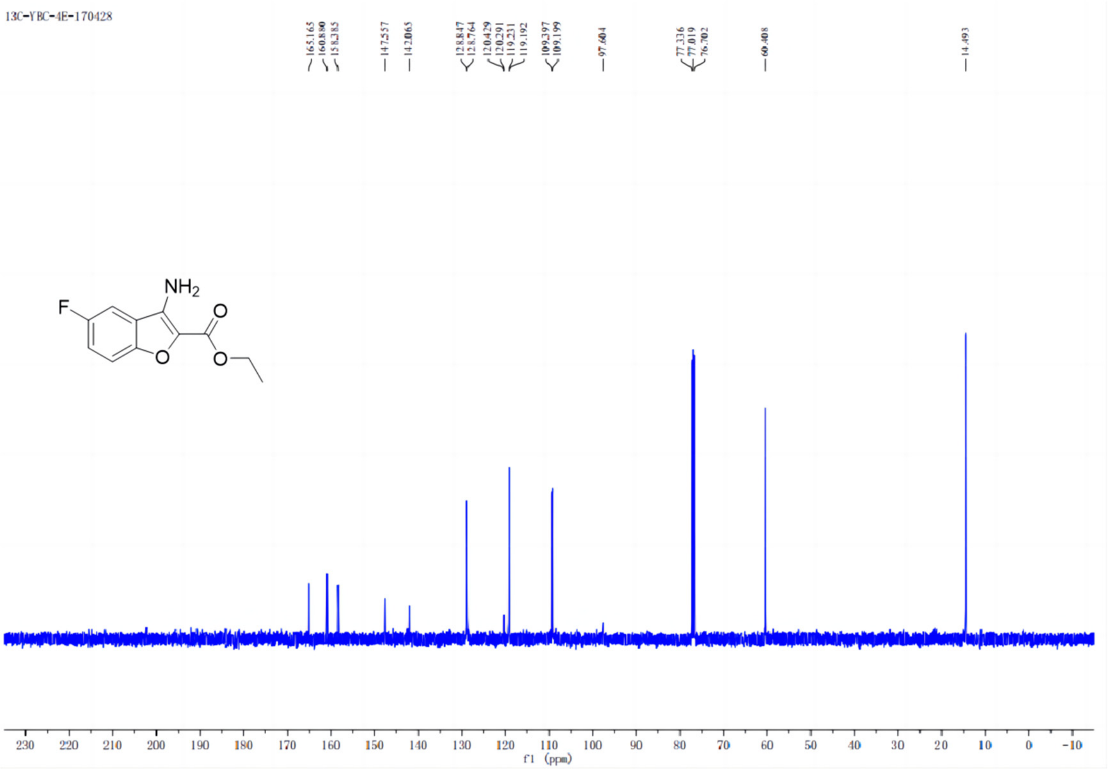

Figure S14. ^{1}H NMR and ^{13}C NMR spectra of compound **3m**

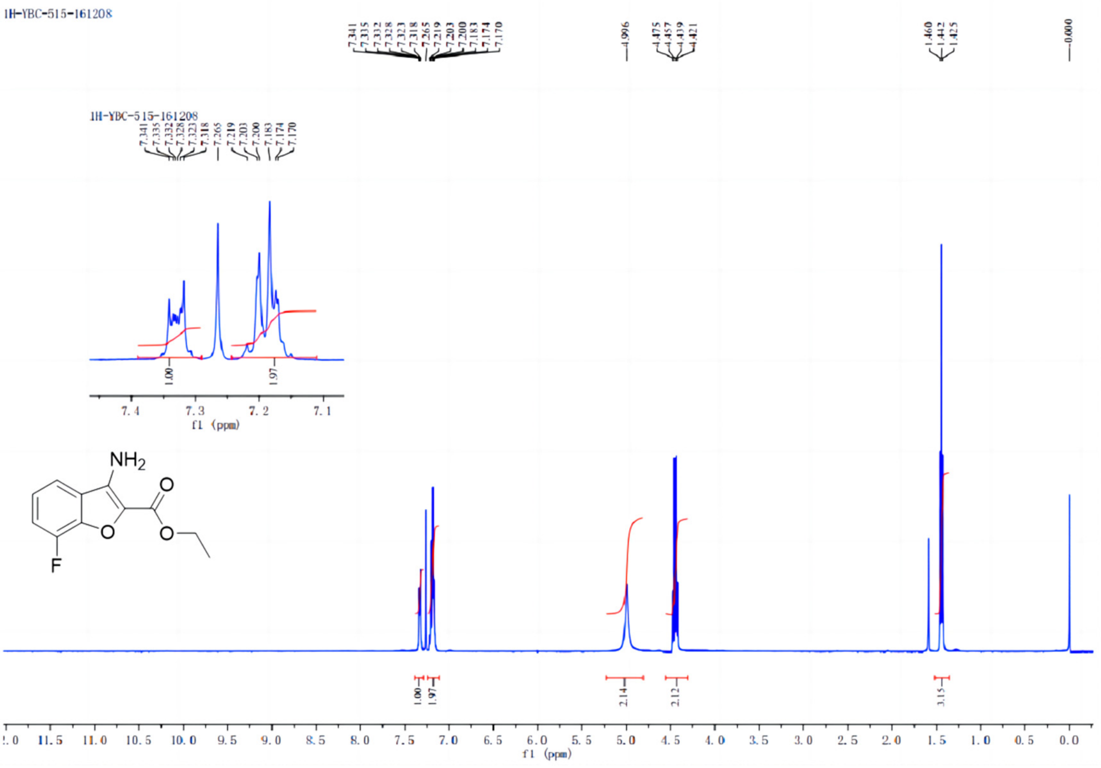

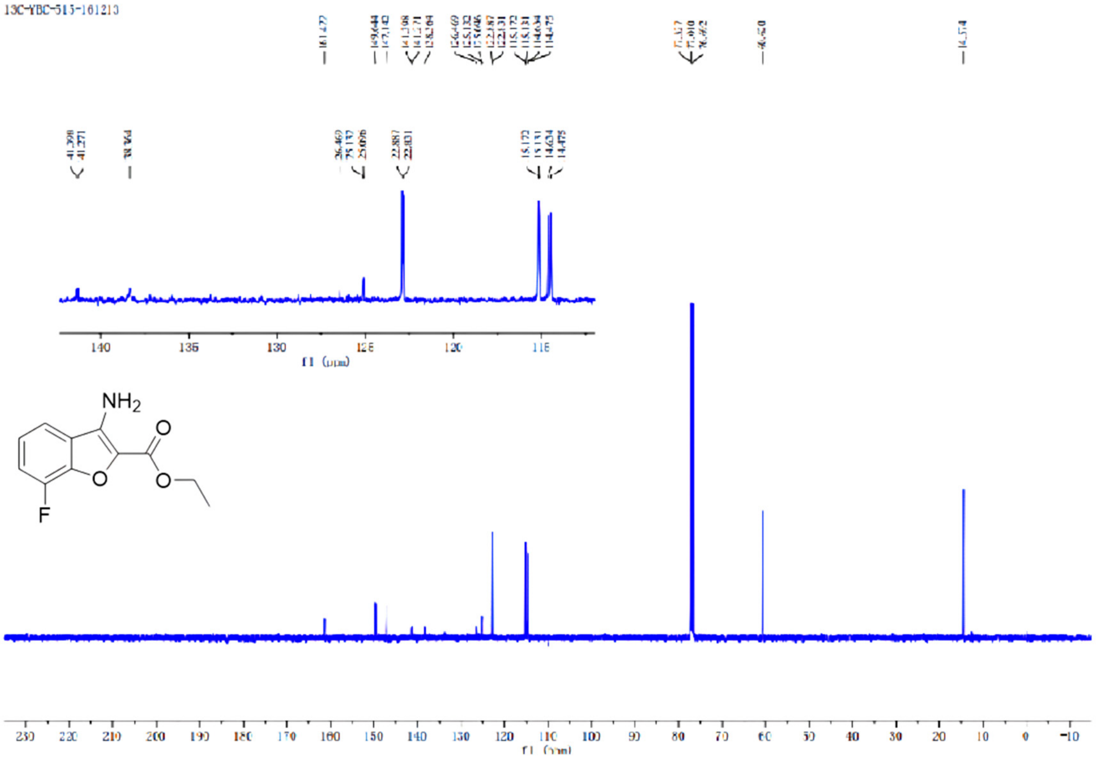

Figure S15. ^{1}H NMR and ^{13}C NMR spectra of compound **3n**

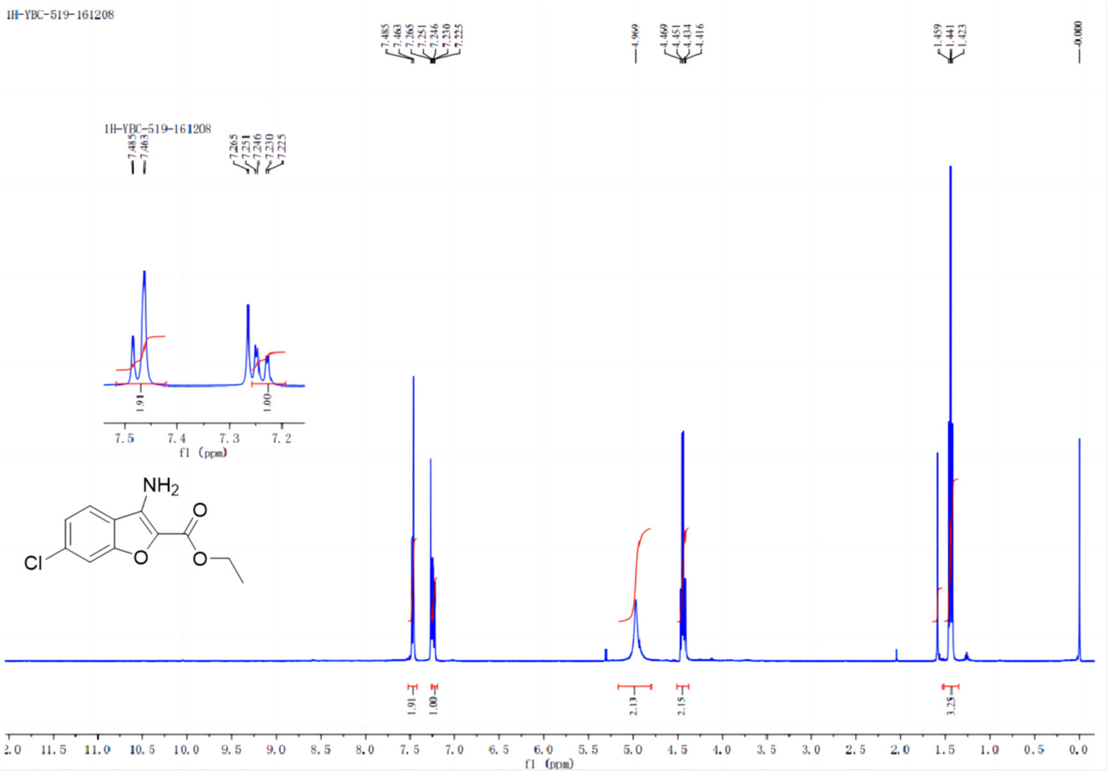

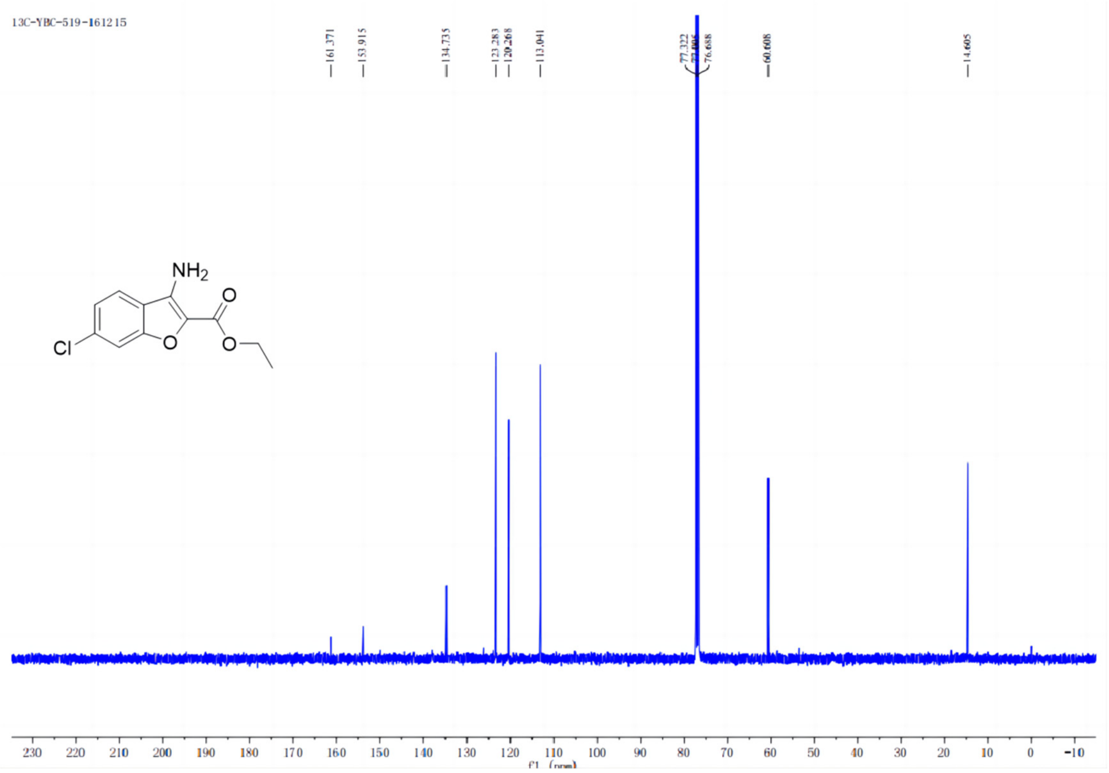

Figure S16. ^{1}H NMR and ^{13}C NMR spectra of compound **3o**

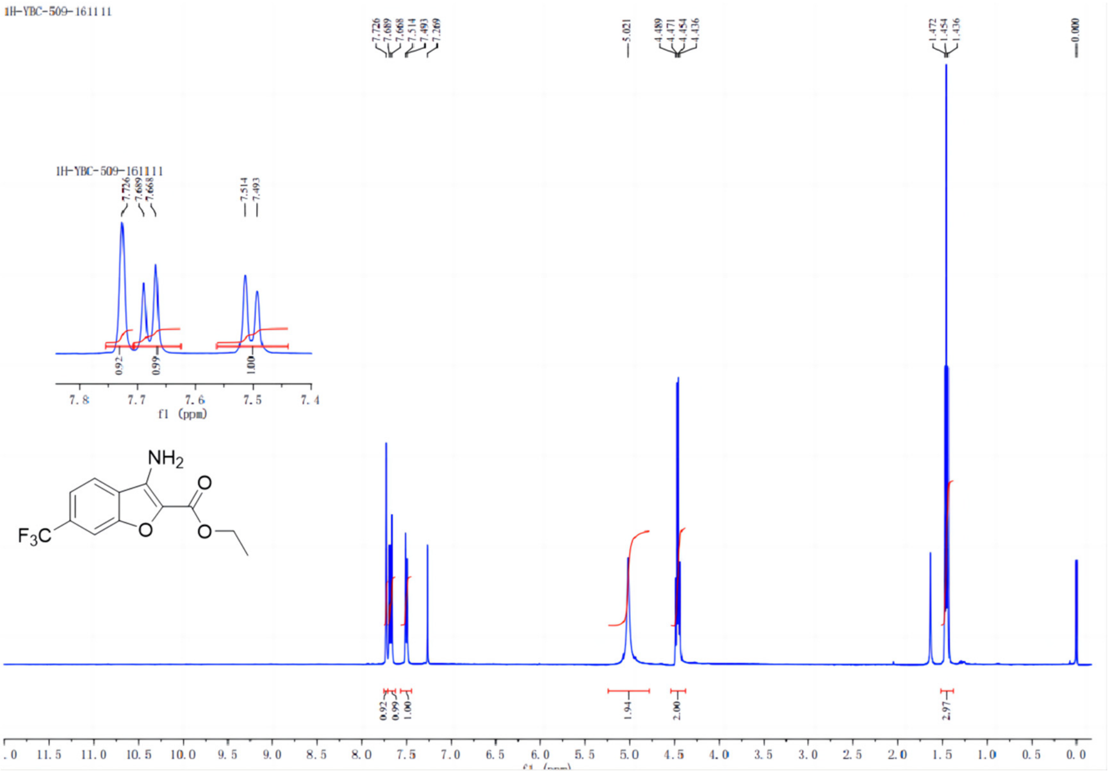

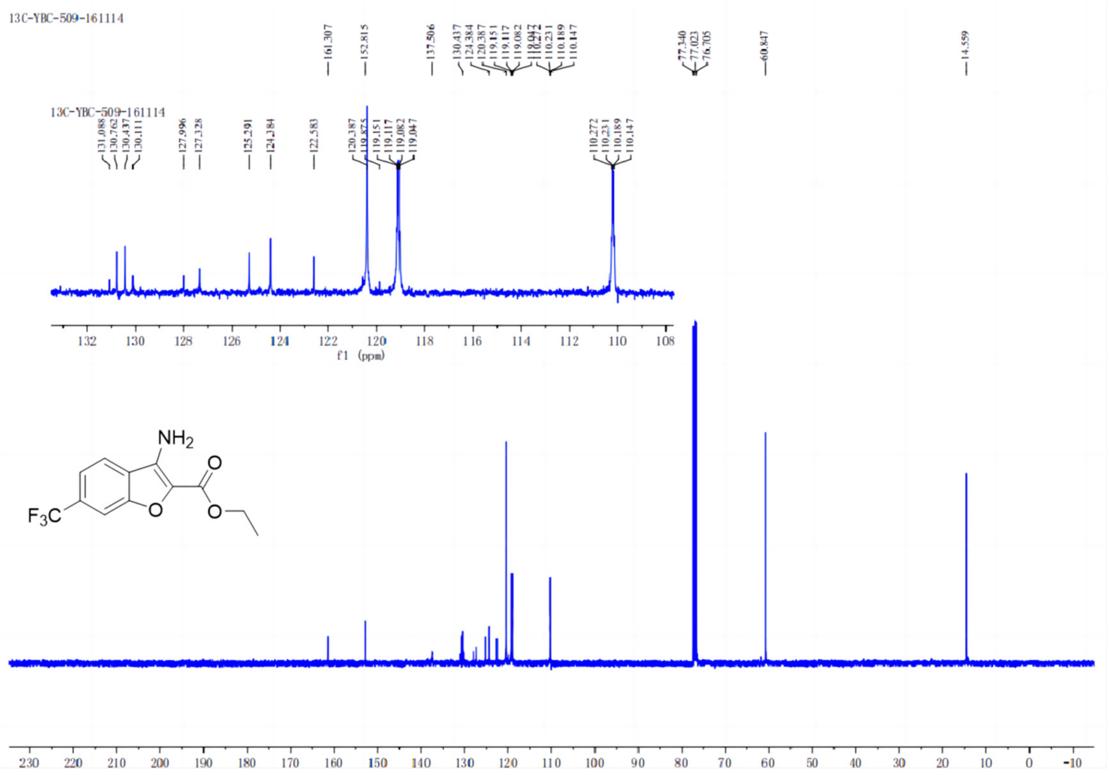

Figure S17. ^{1}H NMR and ^{13}C NMR spectra of compound **3p**

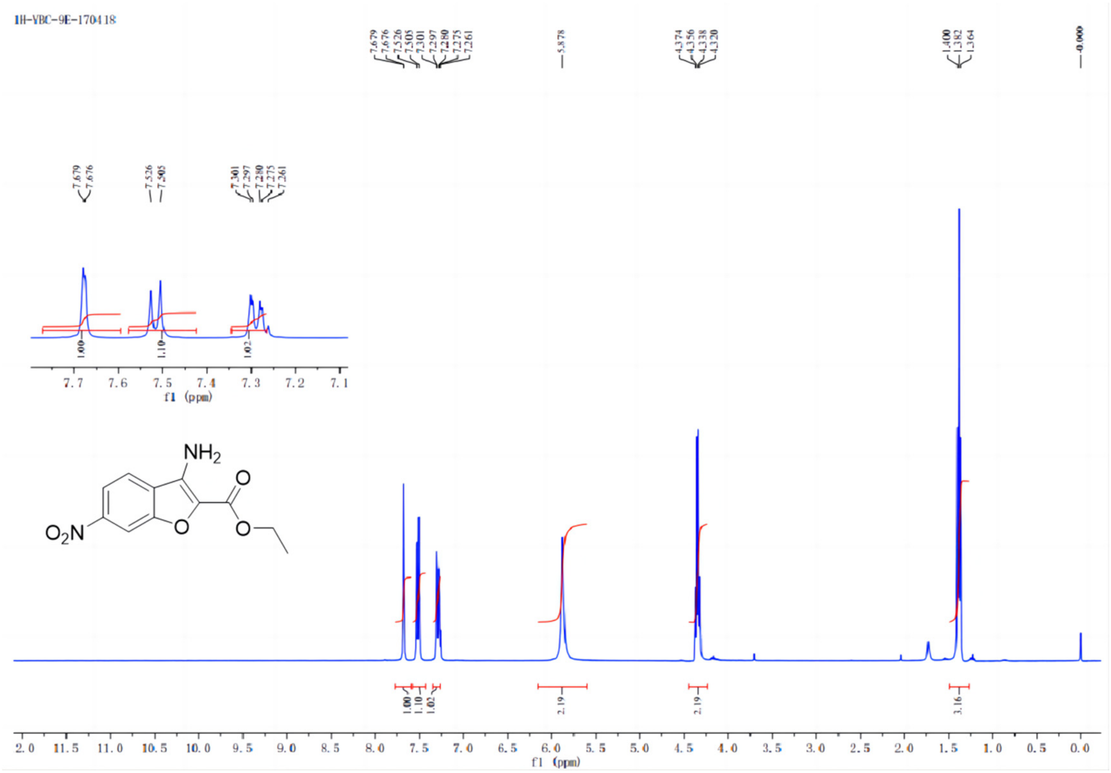

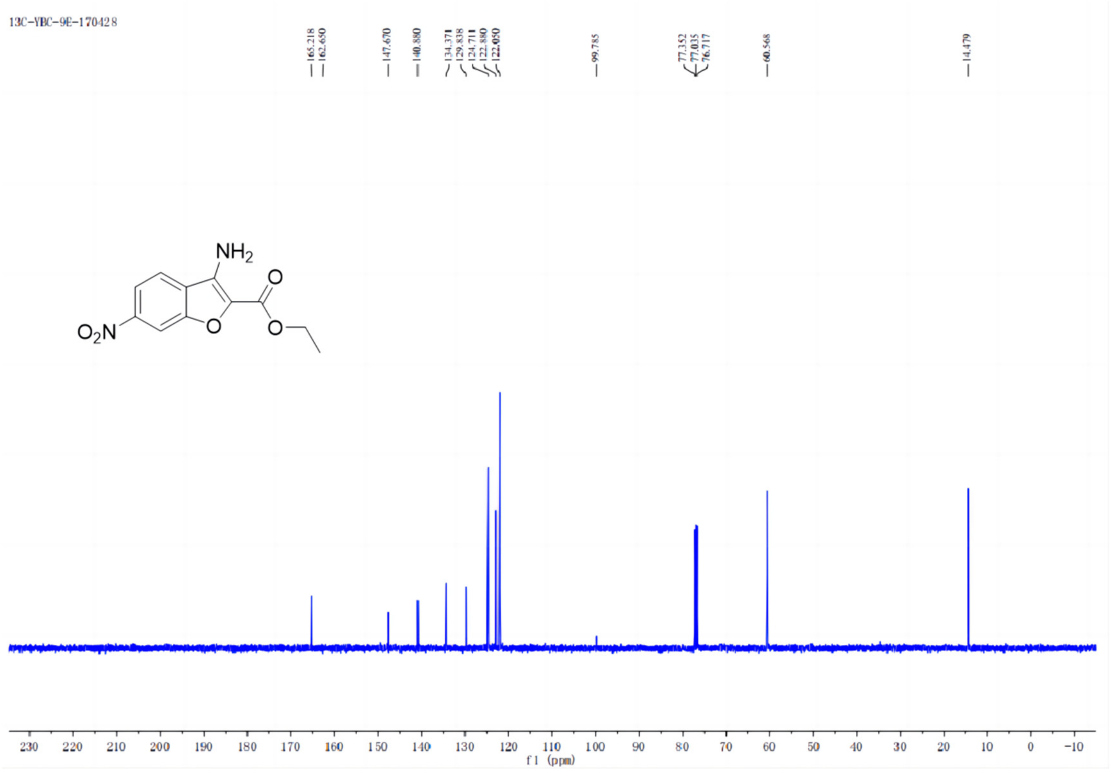

Figure S18. ^{1}H NMR and ^{13}C NMR spectra of compound **3q**

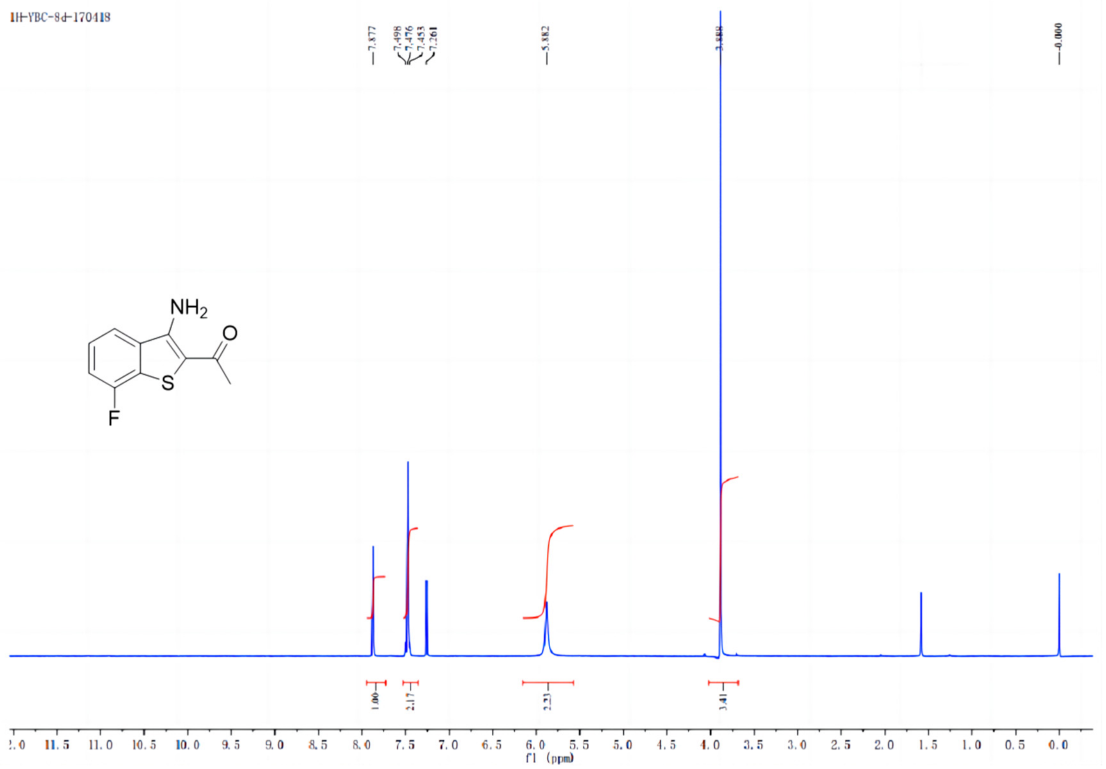

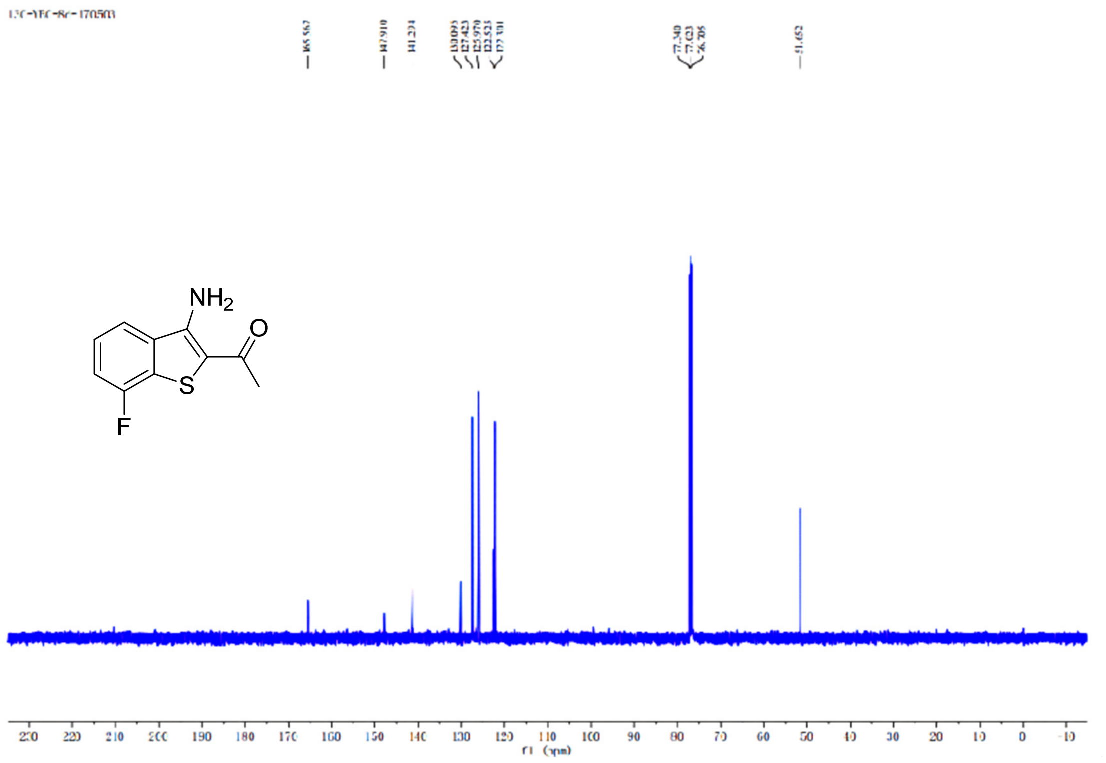

Figure S19. ^{1}H NMR and ^{13}C NMR spectra of compound **5a**

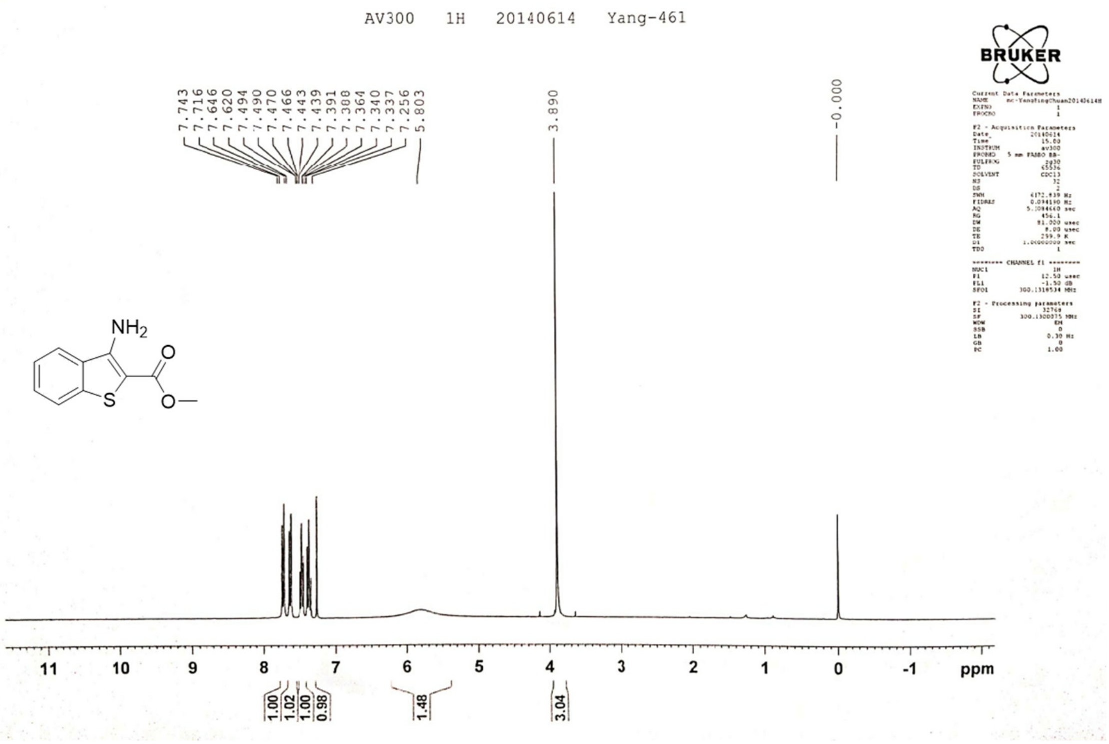

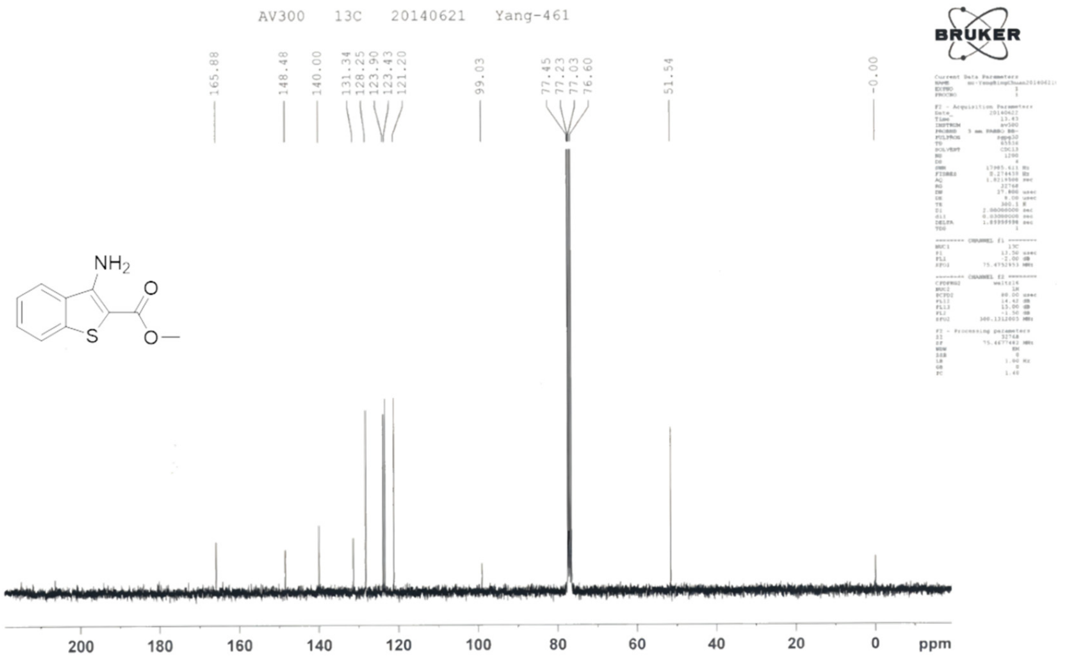

Figure S20. ^{1}H NMR and ^{13}C NMR spectra of compound **5b**

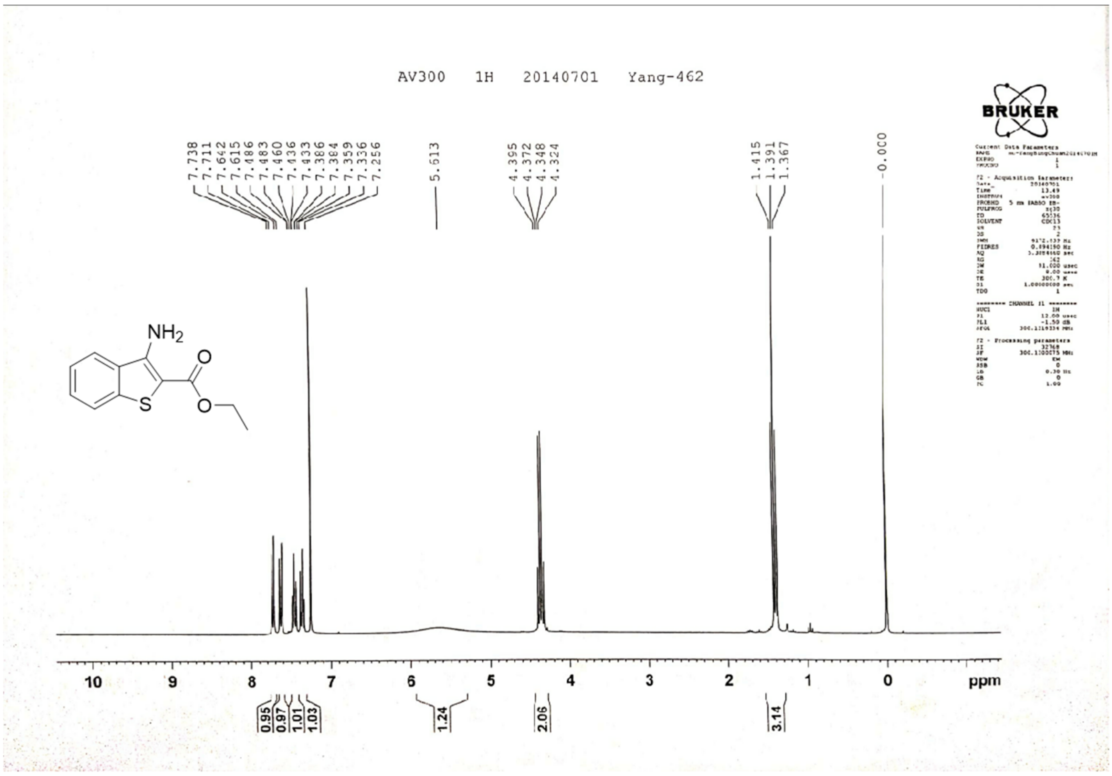

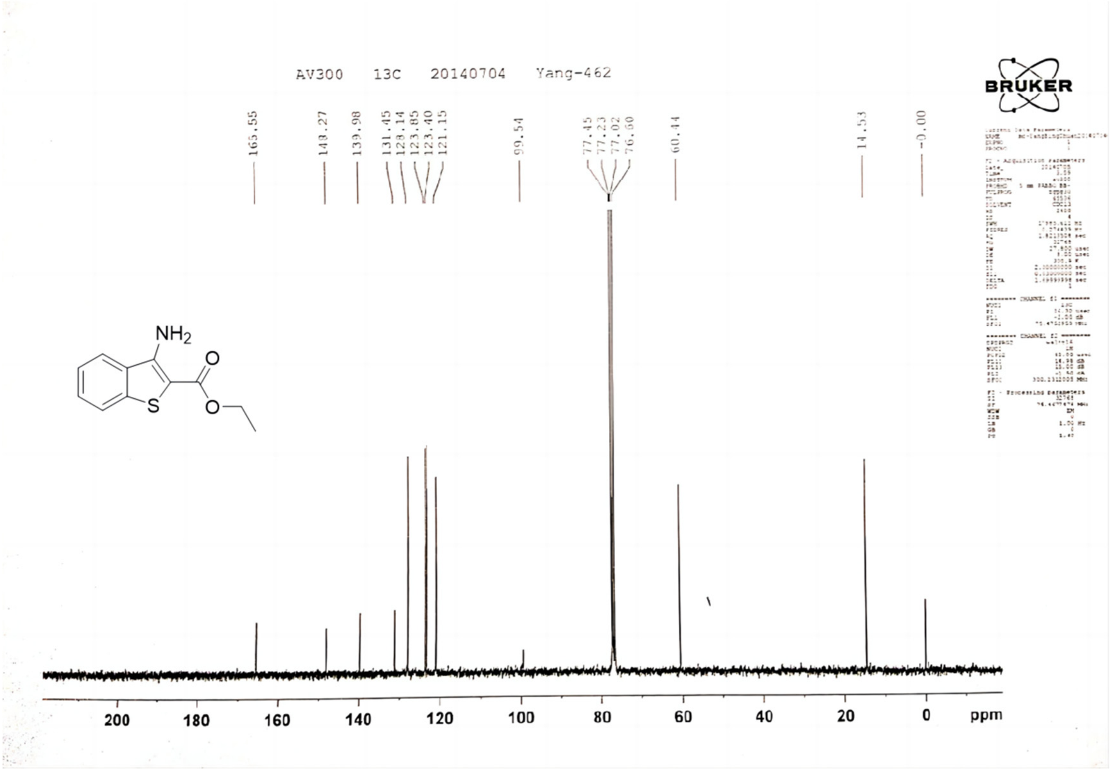

Figure S21. ^{1}H NMR and ^{13}C NMR spectra of compound **5c**

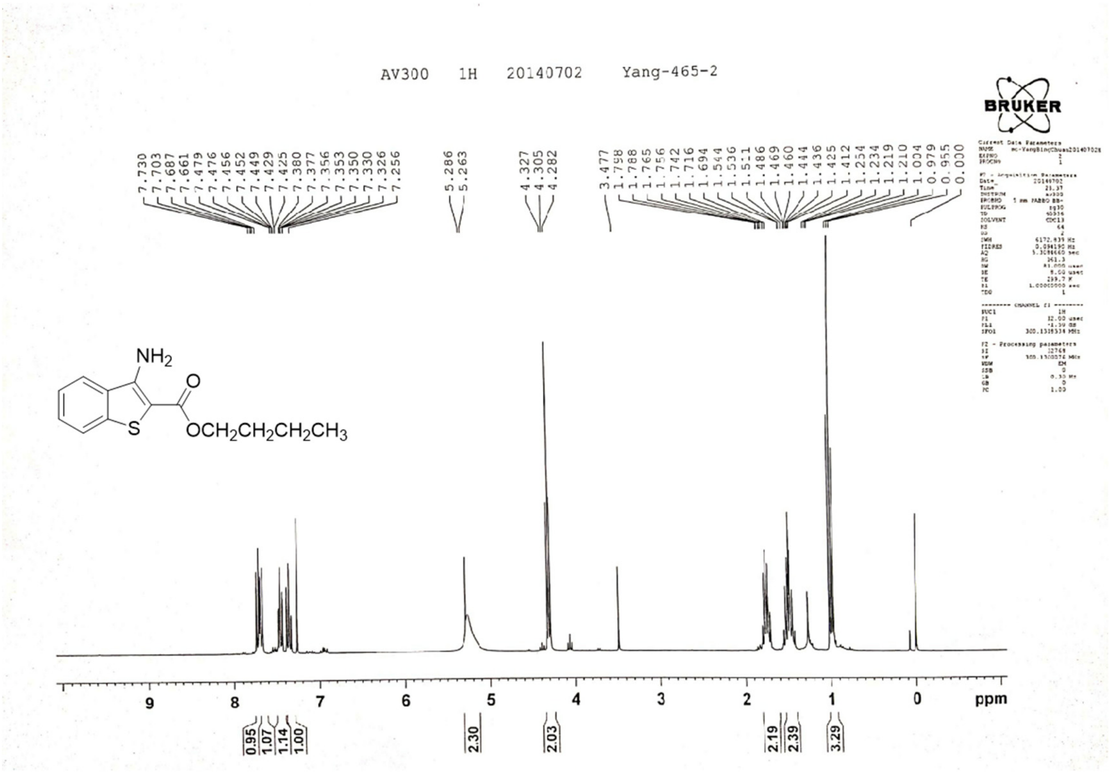

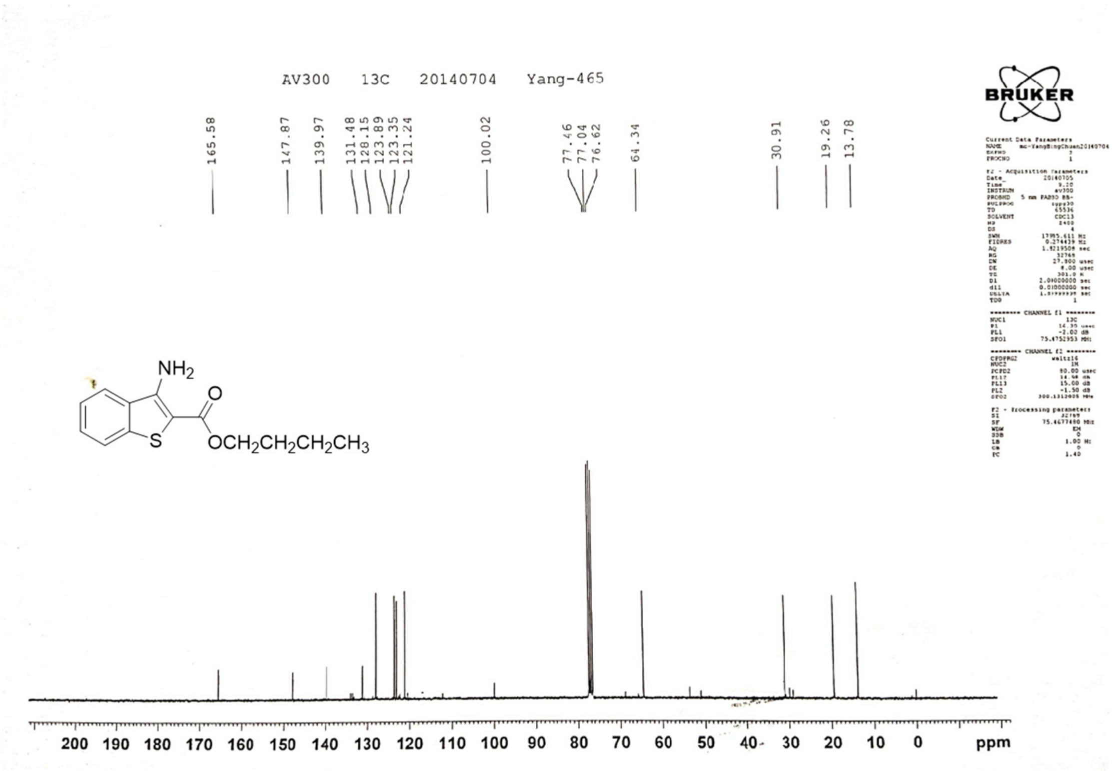

Figure S22. ^{1}H NMR and ^{13}C NMR spectra of compound **5d**

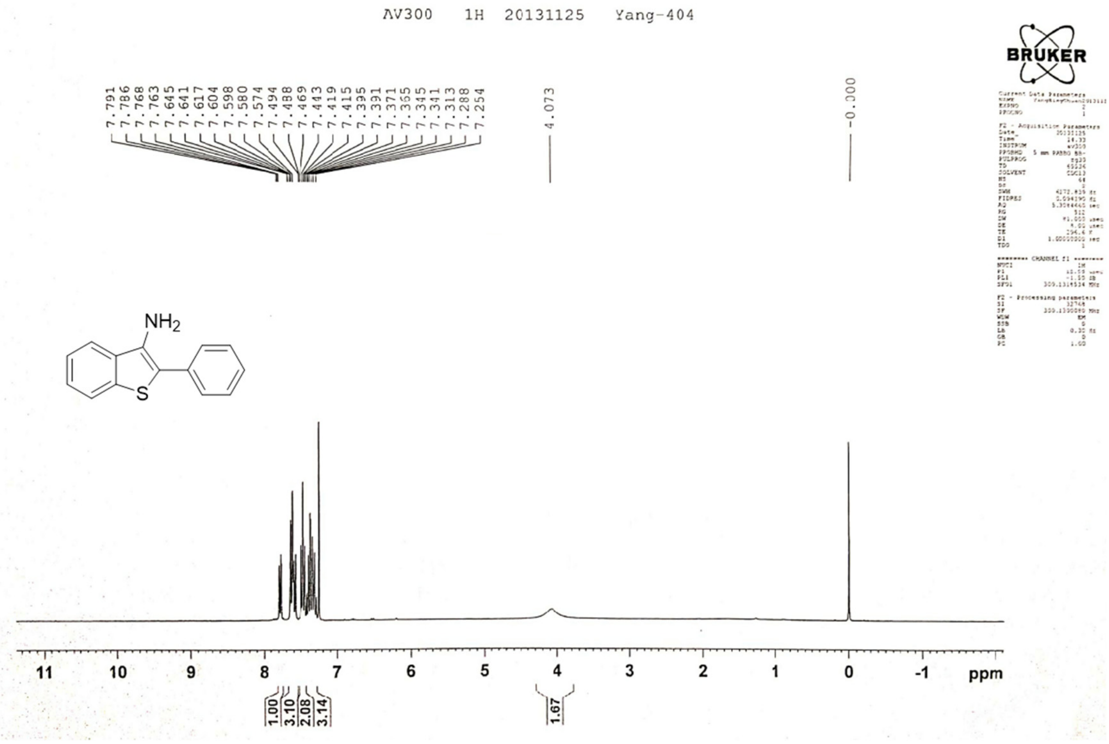

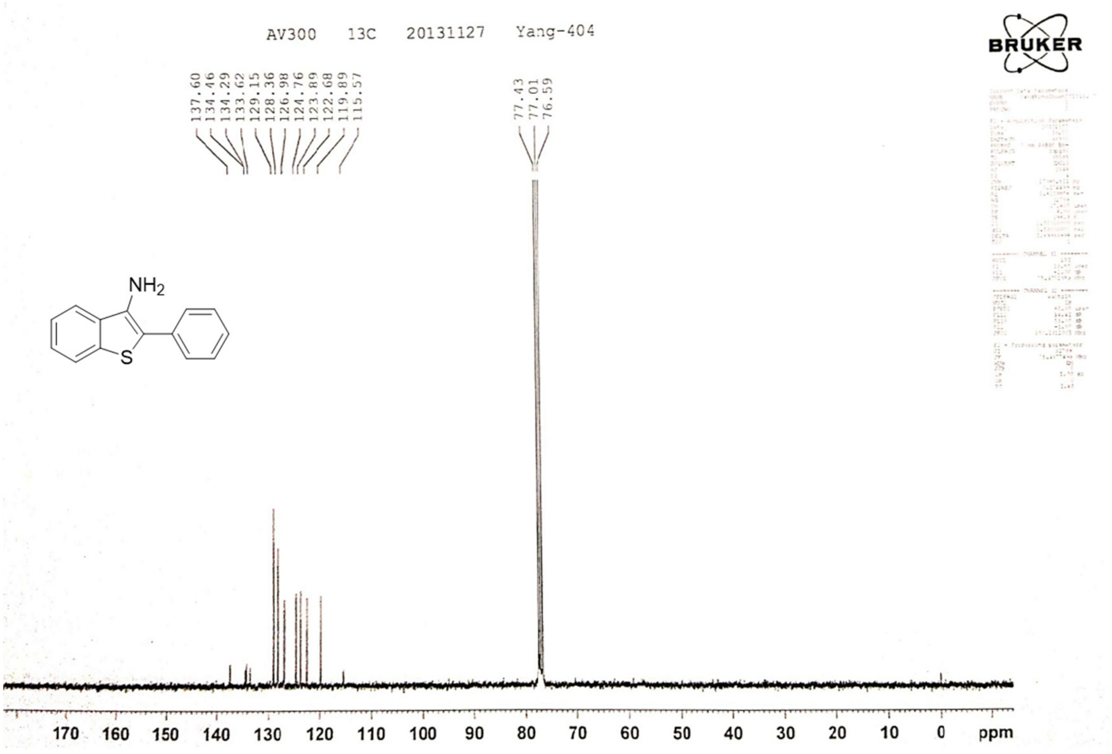

Figure S23. ^{1}H NMR and ^{13}C NMR spectra of compound **5e**

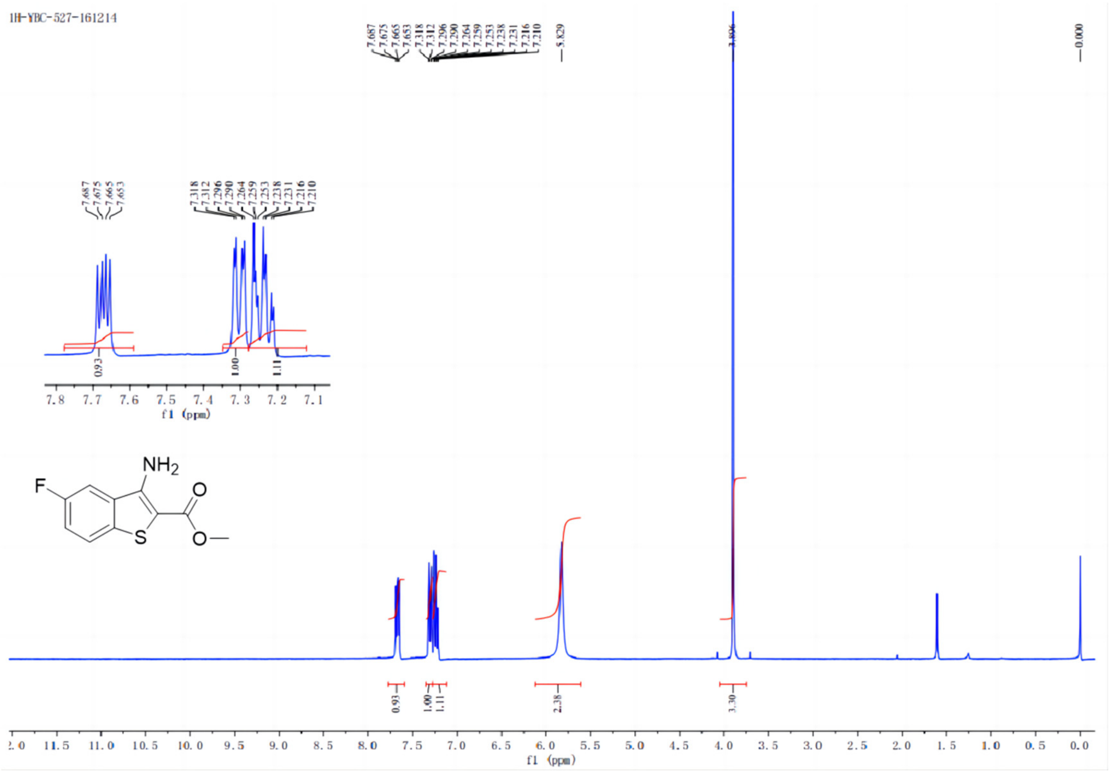

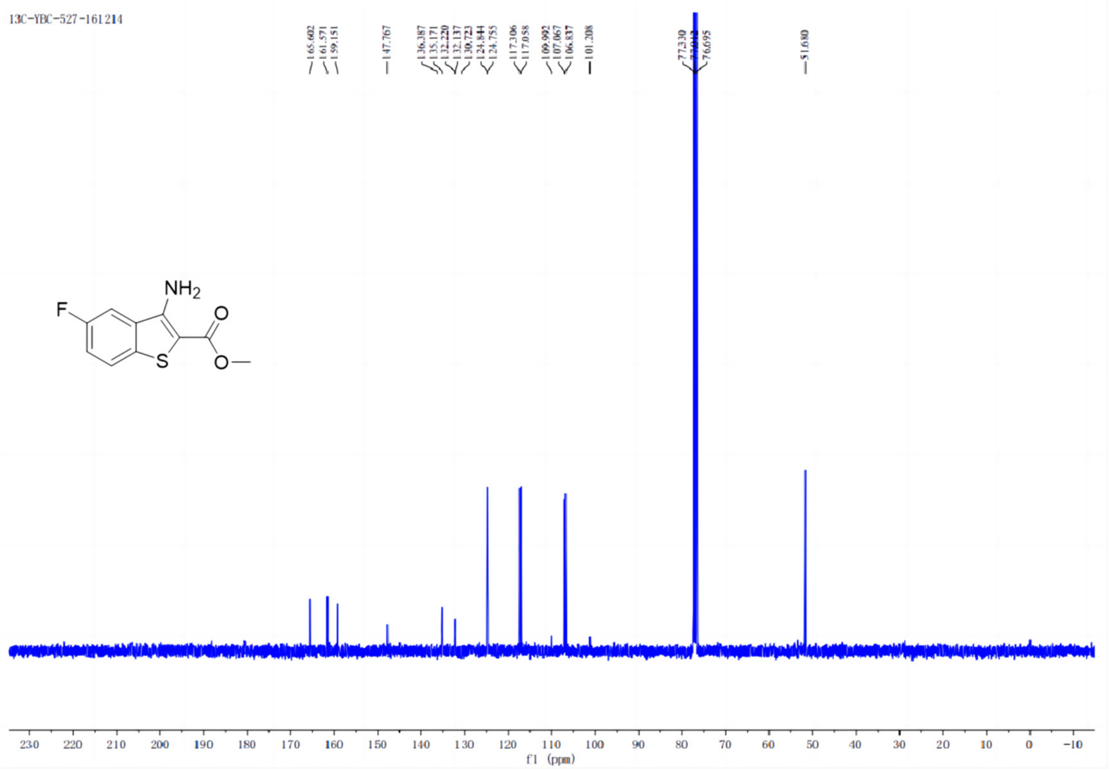

Figure S24. ^{1}H NMR and ^{13}C NMR spectra of compound **5f**

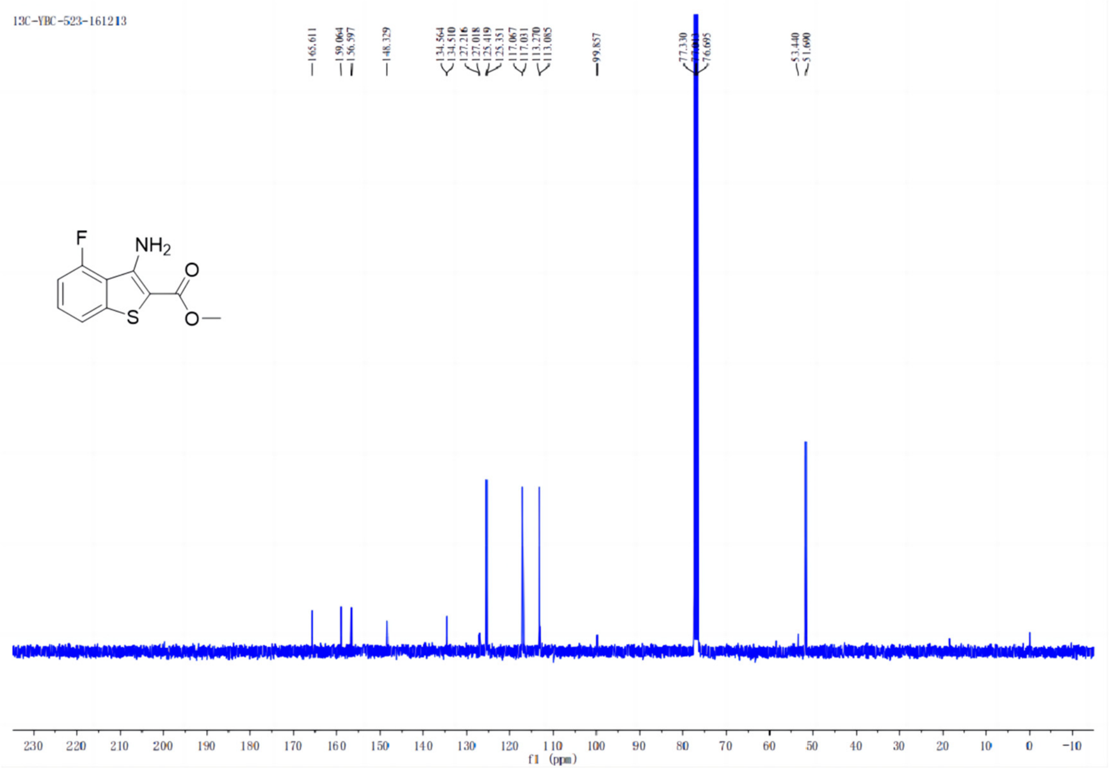

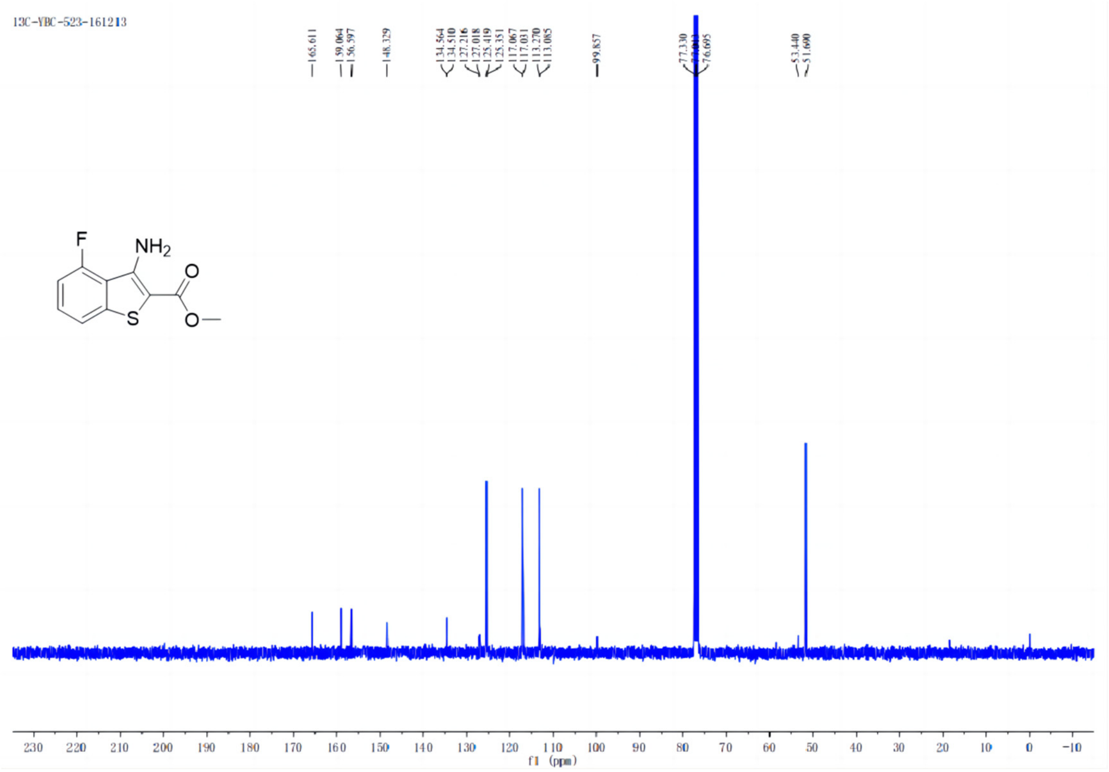

Figure S25. ^{1}H NMR and ^{13}C NMR spectra of compound **5g**

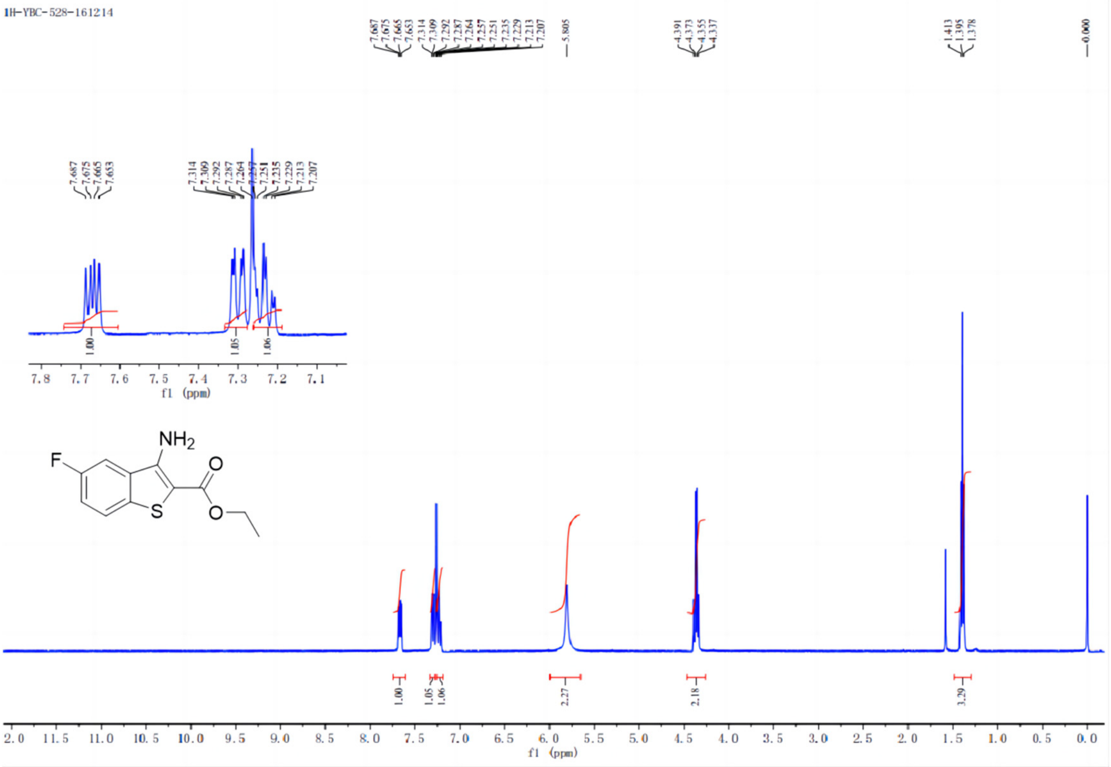

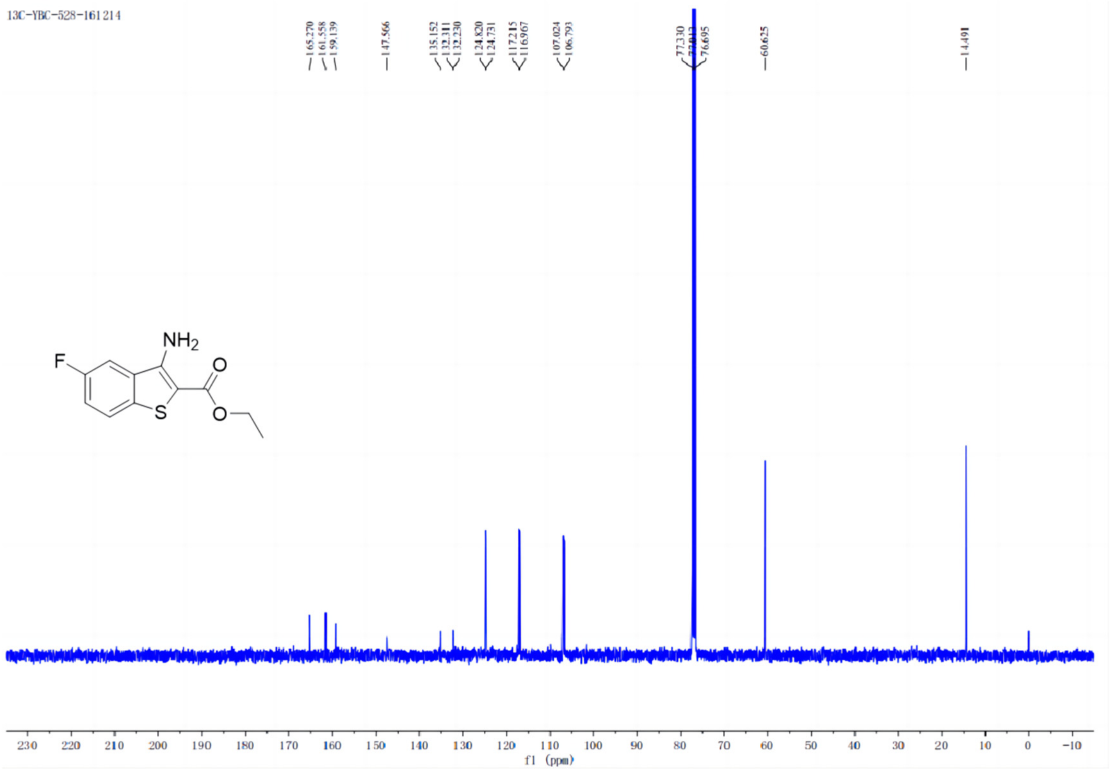

Figure S26. ^{1}H NMR and ^{13}C NMR spectra of compound **5h**

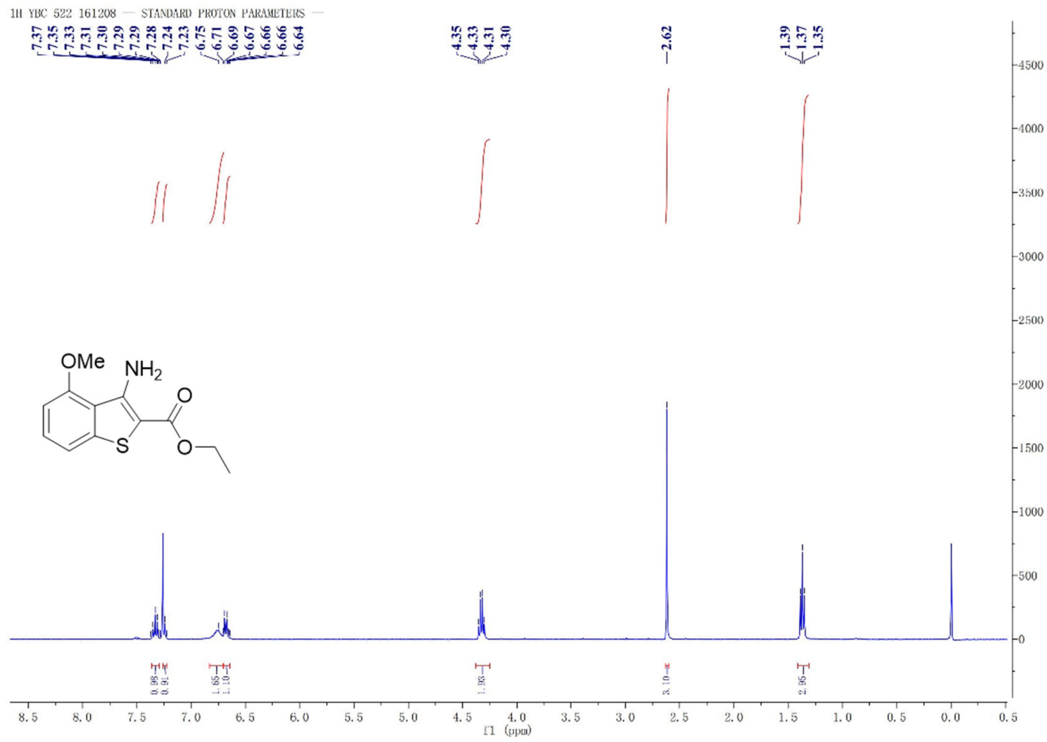

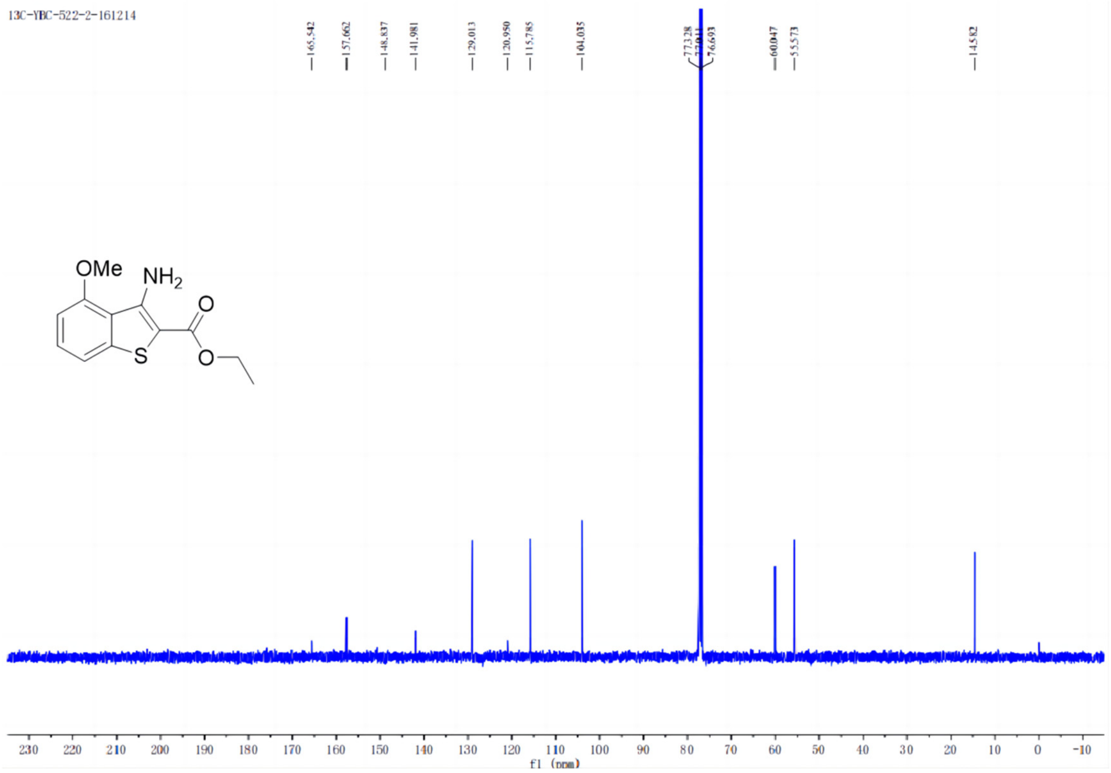

Figure S27. ^{1}H NMR and ^{13}C NMR spectra of compound **5i**
